# Advanced imaging of colorectal cancer: From anatomy to molecular imaging

**DOI:** 10.1007/s13244-016-0465-x

**Published:** 2016-04-30

**Authors:** Roberto García-Figueiras, Sandra Baleato-González, Anwar R. Padhani, Ana Marhuenda, Antonio Luna, Lidia Alcalá, Ana Carballo-Castro, Ana Álvarez-Castro

**Affiliations:** Department of Radiology, Hospital Clínico Universitario de Santiago de Compostela, Choupana s/n, 15706 Santiago de Compostela, Spain; Paul Strickland Scanner Centre, Mount Vernon Cancer Centre, Northwood, Middlesex, England, HA6 2RN UK; Department of Radiology, IVO (Instituto Valenciano de Oncología), C/ Beltrán Báguena, 8, 46009 Valencia, Spain; Department of Radiology, Advanced Medical Imaging, Clinica Las Nieves, SERCOSA, Grupo Health Time, C/ Carmelo Torres 2, 23007 Jaén, Spain; Case Western Reserve University, Cleveland, OH USA; Department of Radiotherapy, Hospital Clínico Universitario de Santiago de Compostela, Choupana s/n, 15706 Santiago de Compostela, Spain; Department of Gastroenterology, Colorectal Cancer Group, Hospital Clínico Universitario de Santiago de Compostela, Choupana s/n, Santiago de Compostela, 15706 Spain

**Keywords:** Colorectal neoplasms, MRI, functional, Perfusion imaging, Molecular imaging, Multimodal imaging

## Abstract

**Abstract:**

Imaging techniques play a key role in the management of patients with colorectal cancer. The introduction of new advanced anatomical, functional, and molecular imaging techniques may improve the assessment of diagnosis, prognosis, planning therapy, and assessment of response to treatment of these patients. Functional and molecular imaging techniques in clinical practice may allow the assessment of tumour-specific characteristics and tumour heterogeneity. This paper will review recent developments in imaging technologies and the evolving roles for these techniques in colorectal cancer.

***Teaching Points*:**

• *Imaging techniques play a key role in the management of patients with colorectal cancer*.

• *Advanced imaging techniques improve the evaluation of these patients*.

• *Functional and molecular imaging allows assessment of tumour hallmarks and tumour heterogeneity*.

## Introduction

Colorectal cancer (CRC) represents one of the most commonly diagnosed cancers worldwide. It is the second most common cause of cancer death in the western world [1]. A multidisciplinary approach to CRC management, which includes the radiologist’s role, and the optimization of screening, biomarker and genomic analysis, imaging evaluation, surgical techniques, and therapies have improved patients’ management and prognosis and have decreased CRC mortality rate by 20 % in the last years [2].

Conventional imaging techniques have clear limitations for the evaluation of important tumour features. For example, 9-10 % of patients with computed tomography (CT)-indeterminate lung and/or liver lesions during radiological staging of CRC had definite metastases [3, 4]. Besides, an increasing importance is being placed on the non-invasive imaging assessment of tumour-specific characteristics [5–8]. Functional and molecular imaging (FMI) techniques have emerged to address these limitations. This paper is focused on the current role of advanced imaging modalities in CRC patient management.

## Anatomical imaging techniques in CRC

### Conventional imaging technique

Conventional imaging techniques play a central role in CRC because they depict relationships of the tumour to surgical landmarks (e.g., the circumferential resection margin in the rectum), the presence of important prognostic features, evaluate tumour response to treatment, and are useful for surveillance after therapy. In the case of rectal cancer (RC), magnetic resonance imaging (MRI) is the best imaging technique for evaluating main factors that affect treatment and prognosis, including tumour length, location from the anal verge, relationship to the peritoneal reflection, T-stage, depth of extramural tumour growth, lymph node (LNs) status, vascular and neural invasion, distance to the mesorectal resection margin, and invasion to adjacent structures [8, 9]. Beside this, the main focus for innovations in medical imaging has been the achievement of excellence in anatomical resolution. To date, imaging techniques allow image segmentation and volumetric model reconstruction with different clinical applications in CRC.

#### Computed tomographic colonography

**Fig. 1:**
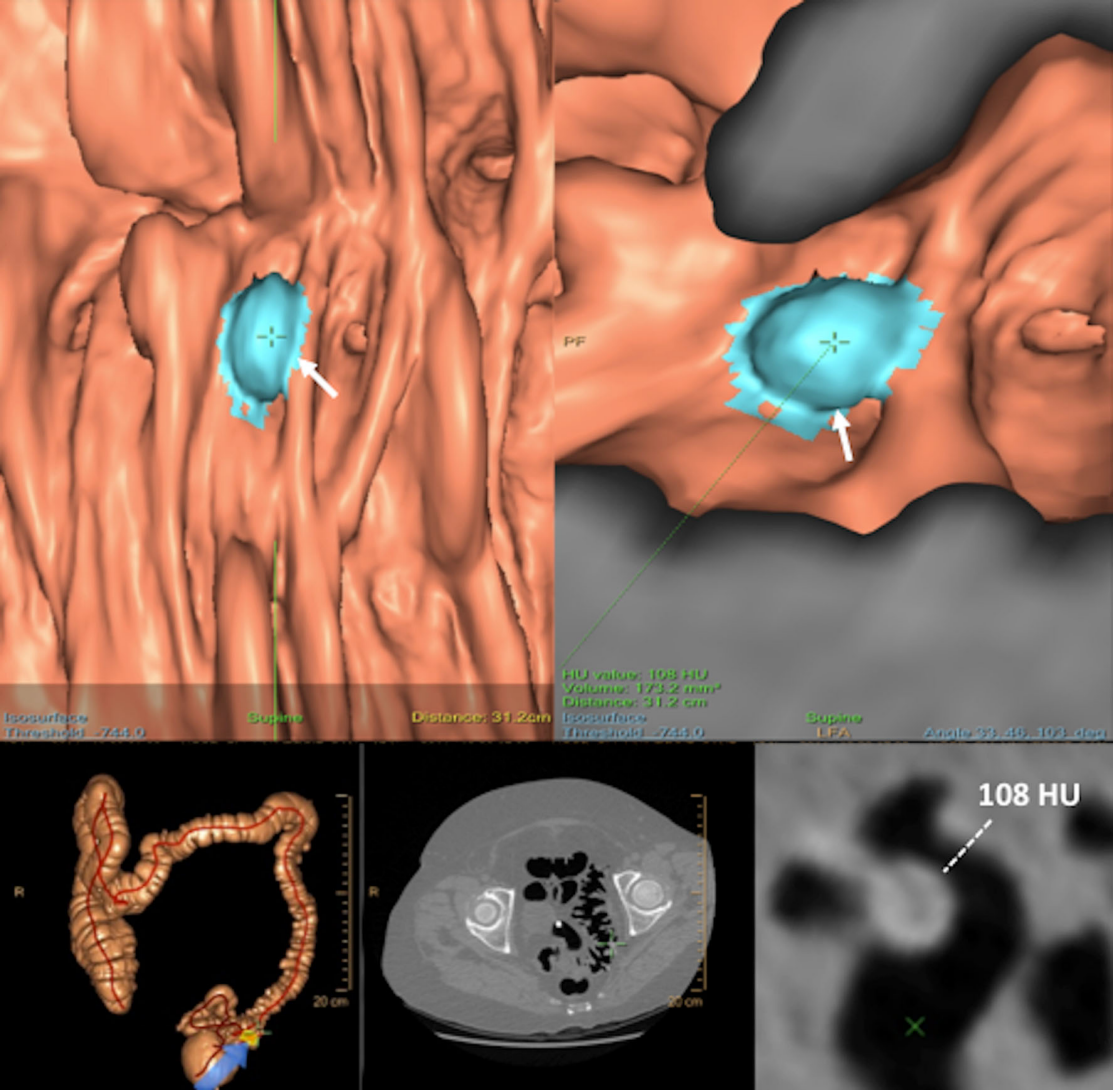
CT colonography in a 40-year-old woman with rectal bleeding. 3D endoluminal virtual dissection view (presenting the bowel as if it has been straightened and unfolded) (top left) and 3D endoluminal view of the colon (top right) showed a suspicious finding (arrows) for the computer-aided polyp detection system (blue lesion). Correlation of these 3D endoluminal views with traditional images at CT demonstrated that this finding corresponded to a high-density (108 HU) impacted diverticulum

Computed tomographic colonography (CTC) involves the use of a CT scanner to produce 2- and 3-dimensional (3D) images of the entire colon and rectum obtained after air insufflation (Fig. 1). CTC can be considered the best radiological diagnostic test for screening CRC and polyps. It has been established that its diagnostic performance for the detection of CRC is similar to that of conventional colonoscopy and is clearly superior to that of a barium enema [10]. Besides, CTC is less invasive than a conventional colonoscopy and easy to perform. Different indications have emerged supported by strong evidence-based data and scientific societies including (1) incomplete, failed, or unfeasible conventional colonoscopy (for diagnosing synchronous cancers), (2) elderly and frail patients (who are more likely to have a complicated colonoscopy), (3) evaluation of alarm symptoms suggestive of CRC, (4) tumour localization (especially for laparoscopic surgery), (5) and evaluation of diverticular disease and of patients with colonic stoma [11]. Other indications, many of which are still being debated, include CRC screening and surveillance after surgery for CRC or polypectomy [11–13].

#### Volumetry in CRC

Tumour sizes and volumes have been proven to be an important prognostic indicator for a variety of tumours. However, these features were not found useful in the TNM staging system in CRC and in predicting the clinical outcome of patients, though published papers are sometimes contradictory [14–16]. Simple methods are available for measuring volume based on different semi-automated techniques. In the case of RC, tumour volume reduction rate (TVRR) following chemoradiotherapy (CRTP) based on T2-weighted volumetry may have a predictive value. TVRR shows a significant correlation with tumour pathological regression grade after preoperative CRTP [17] and a volume reduction ratio >75 % is associated with an increased pathologic complete response rate [18]. The main limitation of T2-weighted images in the restaging of RC post-CRTP is its inability to distinguish between small remaining tumour foci and fibrosis, which impacts negatively on its sensitivity. Moreover, it is difficult to decide which areas remain suspicious for tumours on T2-weighted images and should be included in the volume measurements. Tumour volumetry based on the signal-intensity characteristics of dynamic contrast-enhanced (DCE) or diffusion-weighted (DW) images may be more accurate than conventional T2-weighted images to distinguish between complete and non-complete responders (sensitivity, specificity, accuracy, and area under the curve (AUC) for DCE, DWI, and T2-weighted images, respectively, 86/64/86 %, 73/94/93 %, 79/76/93 %, and 0.76/0.81/0.90 [19]. However, these data need to be considered with caution. Contrast uptake can be prominent in areas with inflammation, altering DCE-based measurements, and susceptibility artifacts and bright areas on high *b*-values images due to the T2 shine-through effect can make the tumour segmentation on DWI not accurate.

**Fig. 2:**
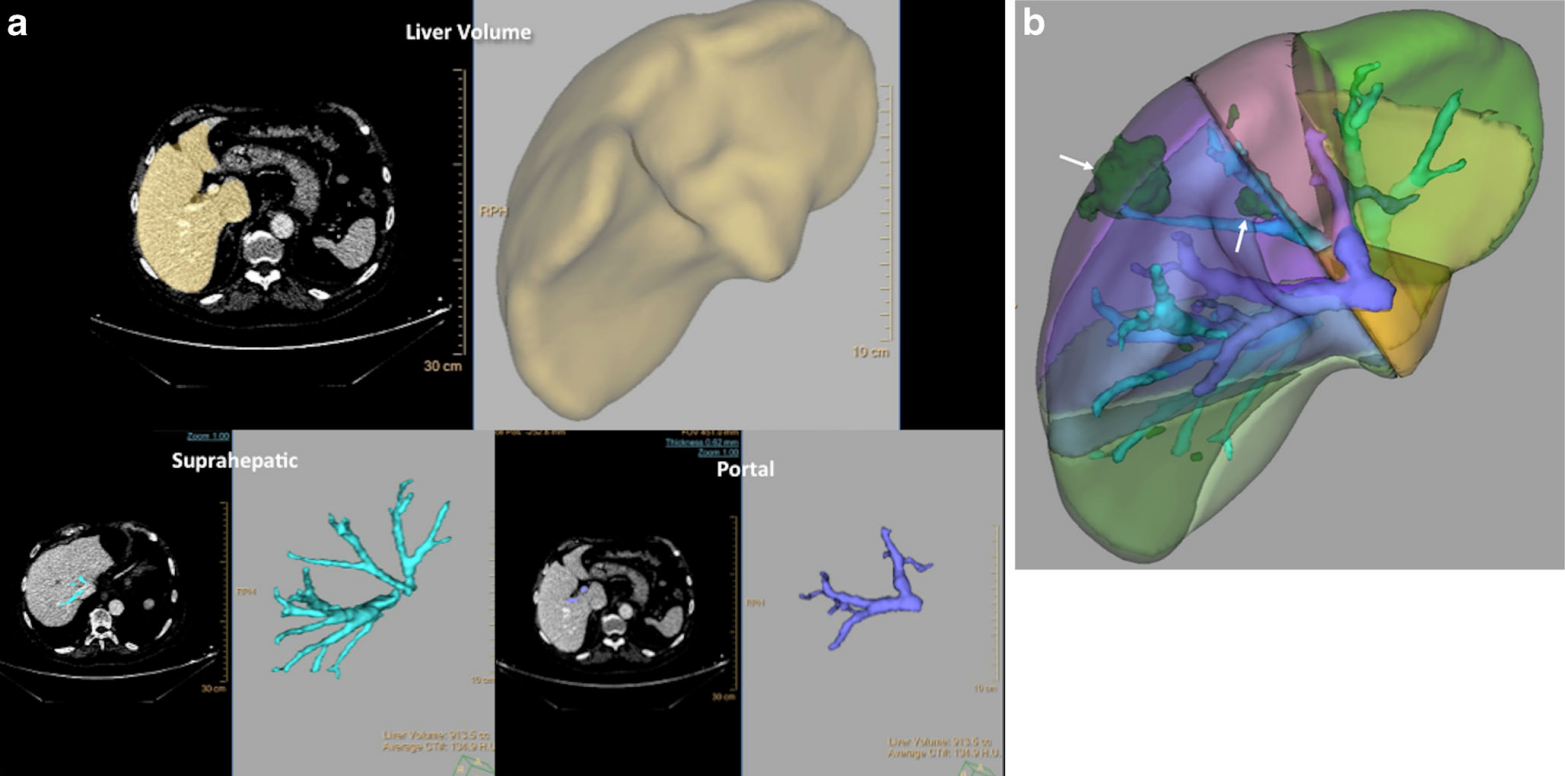
CT volumetry in a patient with colorectal cancer liver metastases. Liver area determined with automated method (yellow) (top-left). Total liver volumetry (1185 cc) (top) and images of the segmentation of the suprahepatic and portal vessels (**a**). Automatic Couinaud liver segmentation using CT images (**b**), which allows the location of the metastatic deposits (green – white arrows)

Hepatic resection has improved the survival of patients with metastatic CRC. Approximately 25 % of newly diagnosed patients with CRC have liver metastases at the time of diagnosis and another 25 % will develop liver metastases during the course of the disease. There are several key features to consider when planning hepatic resection, including the number of segments involved, the proximity of lesions to vascular and biliary structures, and the amount of remnant liver following resection. The size of the remnant liver affects procedural success and postoperative mortality and morbidity [20–22]. This feature is more important in patients with underlying liver disease (e.g., fatty liver secondary to hepatotoxic chemotherapy -CTP- in CRC patients), in which the future liver remnant required needs to be larger than in those patients with a normal liver. Imaging-based liver volumetry has been increasingly utilized to obtain accurate measurements for planning major hepatic resections in patients with CRC [20–22]. Semi-automated computerized liver segmentation methods are mainly based on CT images with the use of liver attenuation for delineating the liver (Fig. 2). However, measurement of attenuation is not possible on MRI. To address this issue, stereology techniques have been used for MRI evaluation with accurate results [23].

### Emerging anatomical imaging techniques

#### Dual-energy computed tomography

**Fig. 3:**
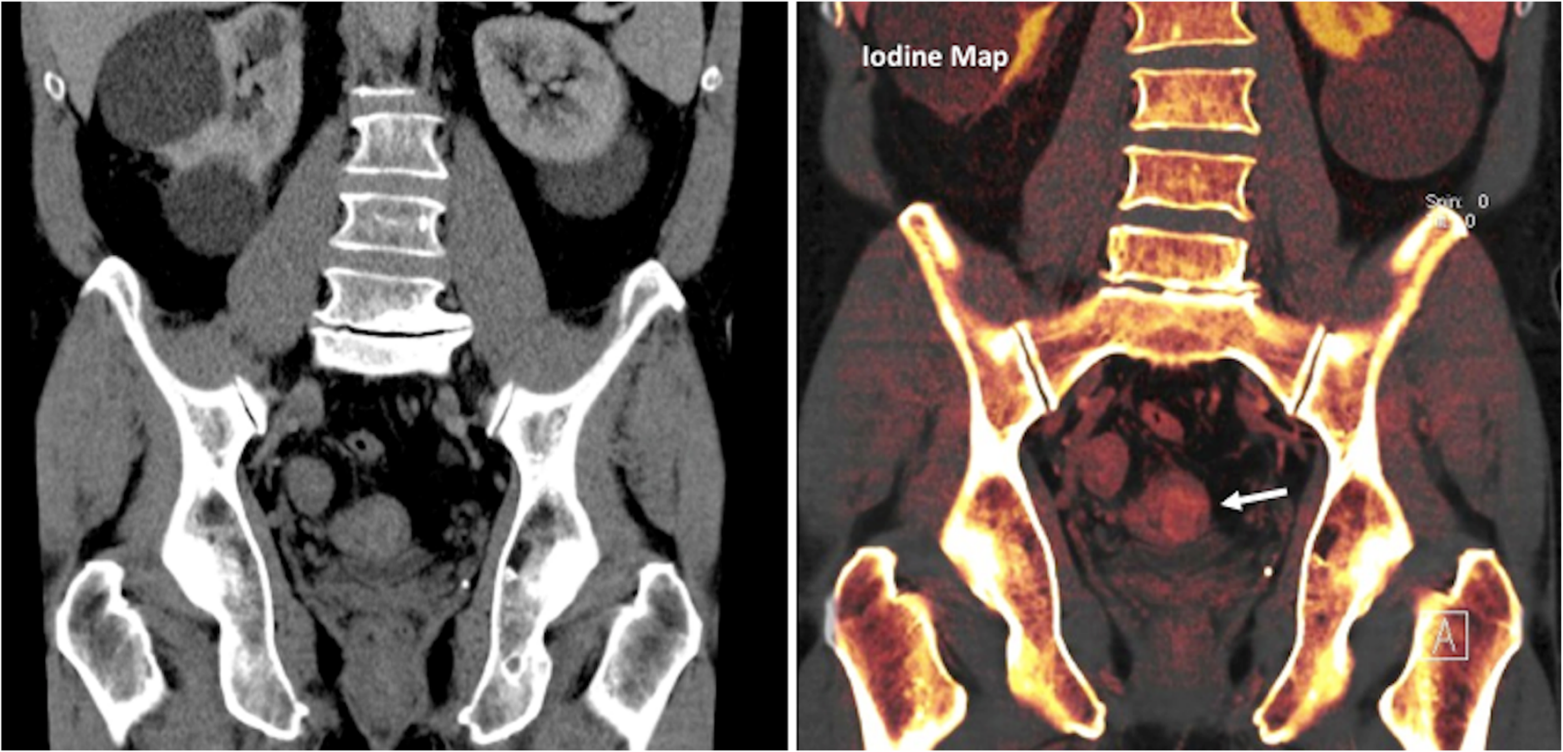
Dual-energy CT in a patient with a rectosigmoid tumour. Coronal reformatted conventional CT (left) and iodine map images. Iodine map depicted iodine uptake in a mass in the sigmoid colon (arrow), improving tumour detection on CT exams without bowel preparation

Dual-energy computed tomography (DECT) is a new technique that allows for differentiation of materials and tissues based on CT density values derived from two synchronous CT acquisitions at different tube potentials in a single acquisition. Iodine uptake can be distinguished from other materials owing to its stronger photoelectric absorption at low tube voltages [24]. DECT improves tissue characterization and material separation. The attenuation caused by iodine on contrast-enhanced CT can be quantified and data can be displayed as a map of iodine concentrations. Additionally, virtual non-enhanced images can also be created. The addition of iodine map evaluation may improve CRCs detection on the contrast-enhanced DECT without bowel preparation compared to only contrast-enhanced images (accuracy 96.7 % vs 90 %) [24] (Fig. 3). DECT may also be useful for tumour staging. The iodine concentration (IC) in the portal phase (PP) had the highest ability to discriminate LN metastasis (AUC 0.932). When clinically obvious metastatic LNs based on conventional CT findings are excluded, the IC in PP remained the most powerful predictor of metastatic LNs (AUC 0.933) [25]. For its part, combining normalized IC in PP with the short axis diameter of LNs, the overall accuracy could be improved to 82.9 % for differentiating metastatic from non-metastatic LNs in RC [26]. Nonetheless, the limitations of these studies include the small sample size, the exclusion of lymph nodes (LNs) less than 2 mm, and an incomplete radiological-histological one-to-one comparison, as not all the LNs identified on pathologic exam were evaluated on CT images.

#### Texture analysis

Malignant tumours display a great spatial and temporal heterogeneity in their biological characteristics and behavior. However, much of the heterogeneity visible on imaging may represent noise. Texture analysis can reduce the effect of noise in images, while enhancing biologic heterogeneity. Texture analysis focuses on the distribution and relationships of grey-level values within images. Texture extracts basic components (i.e., spatial, frequency, etc.) from conventional images, creates a derived set of sub-images, and allows the quantification of different parameters, including entropy, kurtosis, and standard deviation of the pixel distribution histogram [27]. In the case of CRC, texture features have been shown to correlate with KRAS expression, patient’s survival, tumour staging, and tumour response [28–30]. Cui et al. reported that CT texture evaluation of LNs in CRC patients demonstrated a greater heterogeneity (the fraction of pixels that deviate more than a certain range, 10 % default, from the average intensity) in malignant nodes with a size greater than 3 mm and less than 10 mm and fractal dimension [28]. However, in contrast, less-heterogeneous tumours have been associated with a poorer outcome. In this sense, Ng et al. reported that primary CRC at fine filter levels in contrast-enhanced CT were showed a poorer 5-year overall survival (OS) rate with values for entropy, kurtosis, and standard deviation of pixels of less than 7.89, 2.48, and 61.83, respectively, and at least 0.01 for uniformity and −0.38 for skewness [29]. Luebner et al. demonstrated that CRC tumours with hepatic metastases that are more homogeneous at coarse filters (less entropy, smaller standard deviation, higher in attenuation/higher mean of positive pixels) are potentially more aggressive in their biology with higher tumour grade and poorer OS [30]. To explain this apparent contradiction between studies, we must consider the limitations of texture analysis, which include that biologic correlates of texture analysis have not been definitively confirmed in histological studies and that image acquisition parameters and analysis (unenhanced vs contrast-enhanced exam, pattern of contrast administration, texture methods, software platforms, etc.) deeply affect measurement of texture features and change their biological correlation (i.e., texture features may reflect cellular distribution on unenhanced images at a fine scale; they may also reflect the distribution of the contrast agent between the intra- and extravascular extracellular space (EES) on contrast-enhanced images) [29].

## Functional and molecular imaging for the evaluation of tumour hallmarks in CRC

CRC typically show characteristic tumour phenotypic alterations, which are manifestations of genetic changes and metabolic reprogramming, including sustained angiogenesis, limitless replication potential, and altered metabolic pathways (including an increased glycolytic capacity) [5]. Anatomic imaging techniques may be insensitive to mapping the distribution of these tumour-specific characteristics. FMI-derived techniques may help to discriminate these features for clinical decision-making [6, 8] (Table 1).

**Table 1:** Review of main functional and molecular imaging techniques for the evaluation of colorectal cancer

Imaging technique	Biological basis of imaging technique	Evaluation parameters obtained	Pathophysiological correlation	Advantages	Disadvantages
Dynamic contrast-enhanced MRI (DCE-MRI)	Contrast medium uptake rates Transfer rates Extra-cellular volume Plasma volume fraction	• *Qualitative*: evaluation of the type of time/signal intensity curve * *Semiquantitative evaluation*: wash-in; wash-out; time to peak enhancement; etc. * *Quantitative analysis* (*based on mathematical models*): Initial area under gadolinium curve (IAUGC); Transfer and rate constants (K^trans^, k_ep_); Leakage space fraction (ve); Fractional plasma volume (vp)	Vessel density Vascular permeability Perfusion Extravascular space Plasma volume	Low toxicity of contrast agents No ionizing radiation Versatility in pulse sequences	Complex biological explanation of many parameters Complex analysis in quantitative models
Perfusion CT (CTP)	Contrast medium uptake rate in tissues, which is influenced by: • Perfusion & transfer rates • Extra-cellular volume • Plasma volume fraction	• *Qualitative evaluation* of the type of time/signal intensity curve * *Semiquantitative evaluation*: Maximum upslope; Peak enhancement; etc. * *Quantitative analysis* (*based on mathematical models*): Blood flow; Blood volume; Transit time; Permeability, K^trans^	Vessel density Vascular permeability Perfusion Tissue cell fraction Plasma volume	Availability Low cost	Contrast agent toxicity Low sensitivity to contrast agents Exposure to ionizing radiation
Imaging techniques based on water diffusion	Diffusivity of water - Monoexponential analysis (DWI) Perfusion component: Intravoxel incoherent motion (IVIM) Structural complexity: Diffusion kurtosis imaging (DKI) Diffusion tensor imaging (DTI)	• Apparent diffusion coefficient (ADC) • Perfusion fraction (f) • Diffusion (D) Non-Gaussian diffusion coefficient (D) and deviations from normal distribution (K) Mean diffusivity, Diffusion anisotropy indices; Fiber orientation mapping	Tissue architecture: cell density & size, extracellular space tortuosity, gland formation, cell membrane integrity, necrosis Microvessel perfusion Quantifying the non-gaussianity of any distribution and may evaluate membrane integrity Anisotropy of tissue structure	Availability No contrast agents No ionizing radiation	Technical complexity of advanced techniques (IVIM, DKI, and DTI)
MR Spectroscopy Imaging (MRSI)	Contrast medium uptake by macrophages of the lymph nodes	Ratios between metabolites Abnormal Peaks of metabolites Absence of normal metabolites	Analysis of metabolic pathways	Specificity No contrast media	Technical complexity Difficult analysis
MR-Lymphography (USPIO)	Contrast medium uptake by macrophages of the lymph nodes	• Qualitative evaluation: Change/No change of signal in lymph nodes	Function of the reticuloendothelial system	Specificity	Contrast agent availability Spatial resolution for detecting micrometastases Complexity
Positron Emission Tomography (PET)	Different metabolic pathways depending on the radiotracer: 1-Energetic Metabolism Fluorodeoxyglucose (FDG)	• SUV = standardized uptake value (ratio between tracer uptake and homogeneous distribution of the tracer within the patient).	Glucose uptake	Emission directly proportional to concentration of contrast agent High sensitivity Whole-body imaging Relative specificity	High cost Low spatial resolution (1–2 mm) No morphological information Radiation exposure Short half-life in many radiotracers -Very short radionuclide agent half-life No evaluation of permeability Technical complexity
	2-Tumour Proliferation Fluorothymidine (FLT)		Activity of thymidine kinase 1		
	3-Hypoxia (F-MISO), (^64^CuATSM), (F-FAZA)		Uptake is influenced by the O2 level in tissue		
	4-Apoptosis (Annexin-V)		Exposure of phosphatidylserine in the cell membrane		

^64^CuATSM = ^64^Cu-diacetyl-bis(N4-methylthiosemicarbazone; ^18^F-FAZA = ^18^F-fluoroazomycin-arabinozide; FDG = Fluorodeoxyglucose; ^18^F-FMISO = ^18^F-fluoromisonidazole; FLT = Fluorothymidine; SUV = standardized uptake value

### Imaging of tumour angiogenesis in CRC

Angiogenesis is a prognostic feature in CRC that has been correlated with important tumour characteristics such as grade and stage and with an increased incidence of metastases and local tumour recurrence. Functional imaging techniques may provide additional insights into the tumour microenvironment. Main imaging techniques for assessing tumour vascularization in the clinical field are DCE techniques based on MRI and CT. These techniques acquire a series of images through a region of interest before, during, and after the intravenous injection of a contrast media [31, 32]. Different method of analysis can be used to evaluate the obtained data, from purely qualitative assessment to complex mathematical modeling. However, not all the studies have shown agreement concerning the capability of imaging to reflect angiogenesis in CRC [33]. Published data related to the correlation of DCE-derived perfusion parameters with morphologic, angiogenic, and molecular prognostic factors in RC results are sometimes contradictory [8, 34–37]. However, DCE techniques may show clinical value in diagnosis, prognosis, planning therapy, assessment of response to treatment, and detection of tumour relapse in CRC patients [6–8, 34, 35].

#### MR-based imaging techniques

**Fig. 4:**
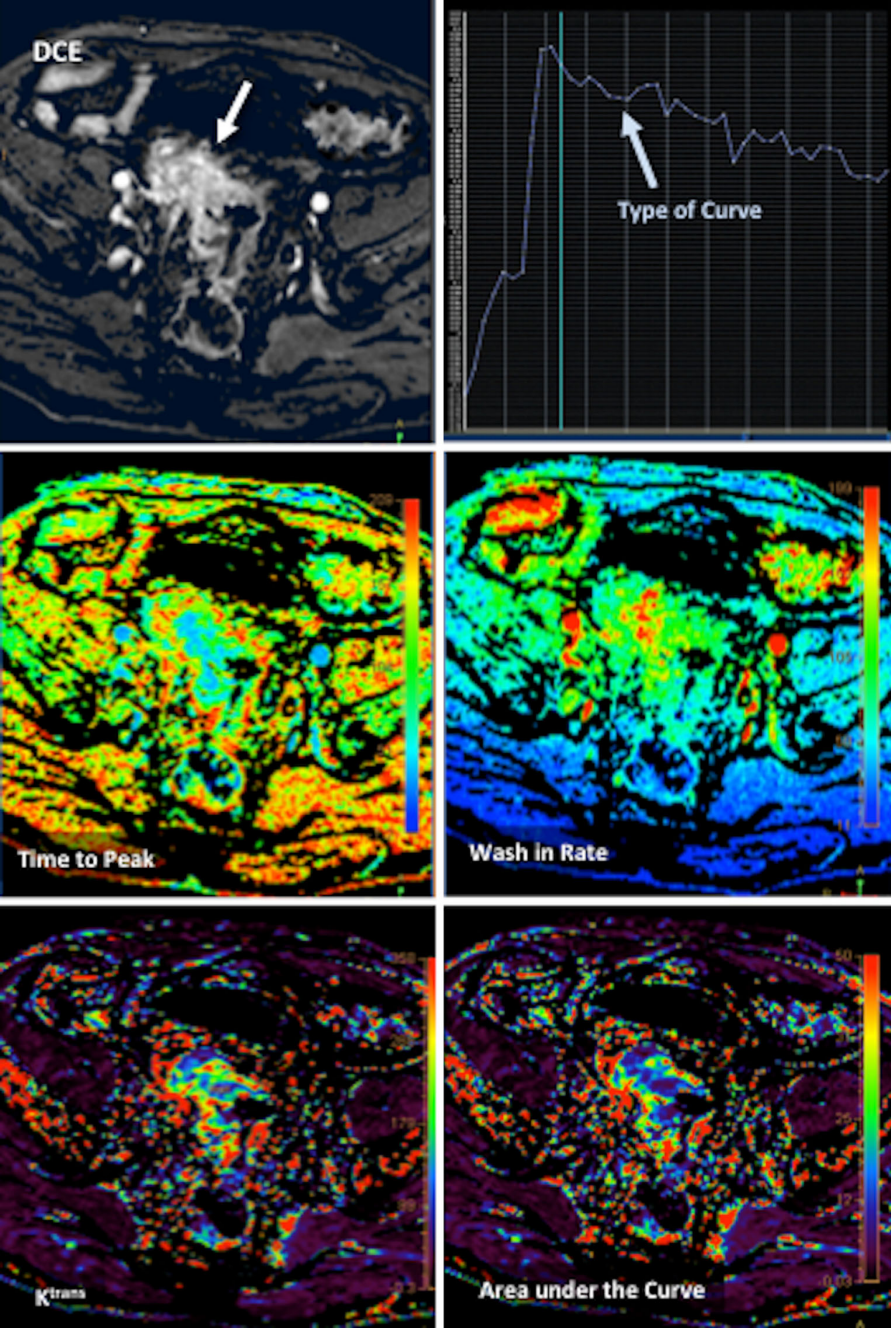
Dynamic contrast-enhanced (DCE) MRI evaluation of a rectal malignant tumour (white arrow) with different types of parameters. Data analysis of DCE-MRI data may be based on different approaches. A qualitative evaluation is based on the visual assessment of tumour enhancement (top left) or type of curve enhancement (top right). Semiquantitative parameters can characterize the shape and structure of the curves of enhancement (middle row). Finally, a quantitative approach is able to measure physiological parameters (bottom)

T1-weighted DCE-MRI techniques are able to evaluate tissue perfusion and vascular leakage based on signal changes secondary to the presence of low-molecular-weight contrast media in the EES [6–8, 31] (Fig. 4). Different parameters can be obtained depending on the complexity of the analytic model applied. Quantitative parameters such as the transfer constant (K^trans^) have demonstrated a prognostic value. Lim et al. reported that K^trans^ values in rectal tumours at presentation in the downstaged group following CRTP were significantly higher than those in the non-downstaged group [38]. In contrast, Gollub et al. in a similar study did not find that pretreatment K^trans^ had predictive value, though post-treatment K^trans^ in a select group was able to distinguish pathological complete responders from incomplete responders [39]. However, different therapies were used in both studies, which could explain these differences. CRTP was used in Lim’s study while CTP/antiangiogenic therapy (bevacizumab) was administrated in Gollub’s study (which may cause a more effective devascularization of the tumour, resulting in lower K^trans^ values). DCE-MRI with the use of blood pool contrast-agents has shown a good performance in RC prognosis of tumour response to CRTP. The late slope was able to discriminate between good and poor responders with an AUC of 0.90, sensitivity of 92 %, specificity of 82 %, positive predictive value (PPV) of 80 %, and negative predictive value (NPV) of 93 % [40]. Evaluation of tumour pathological response after CRTP is another important role of imaging. In this setting, the relative change in K^trans^ has shown a good predictive potential using a cutoff value of 32 % reduction in median K^trans^ with a PPV of 100 % for good response (complete pathologic response -pCR- and near-pCR based on the Mandard’s tumour regression grade) [41], and Tong et al. reported that a K^trans^ threshold of 0.66 could distinguish between complete and incomplete response before CRTP in RC with a sensitivity of 100 % [42]. Finally, DCE-MRI findings correlate with clinical outcome in patients who undergo surgical treatment for recurrent RC. A higher value correlated directly with a complete tumour-free resection margin, while in the case of the rate constant or k_ep_ there was a negative correlation [43]. However, it must be considered that clinical comparability across perfusion analysis solutions is currently not warranted. A considerable variability for DCE-MRI pharmacokinetic parameters has been found among various commercially available perfusion analysis solutions [44].

**Fig. 5:**
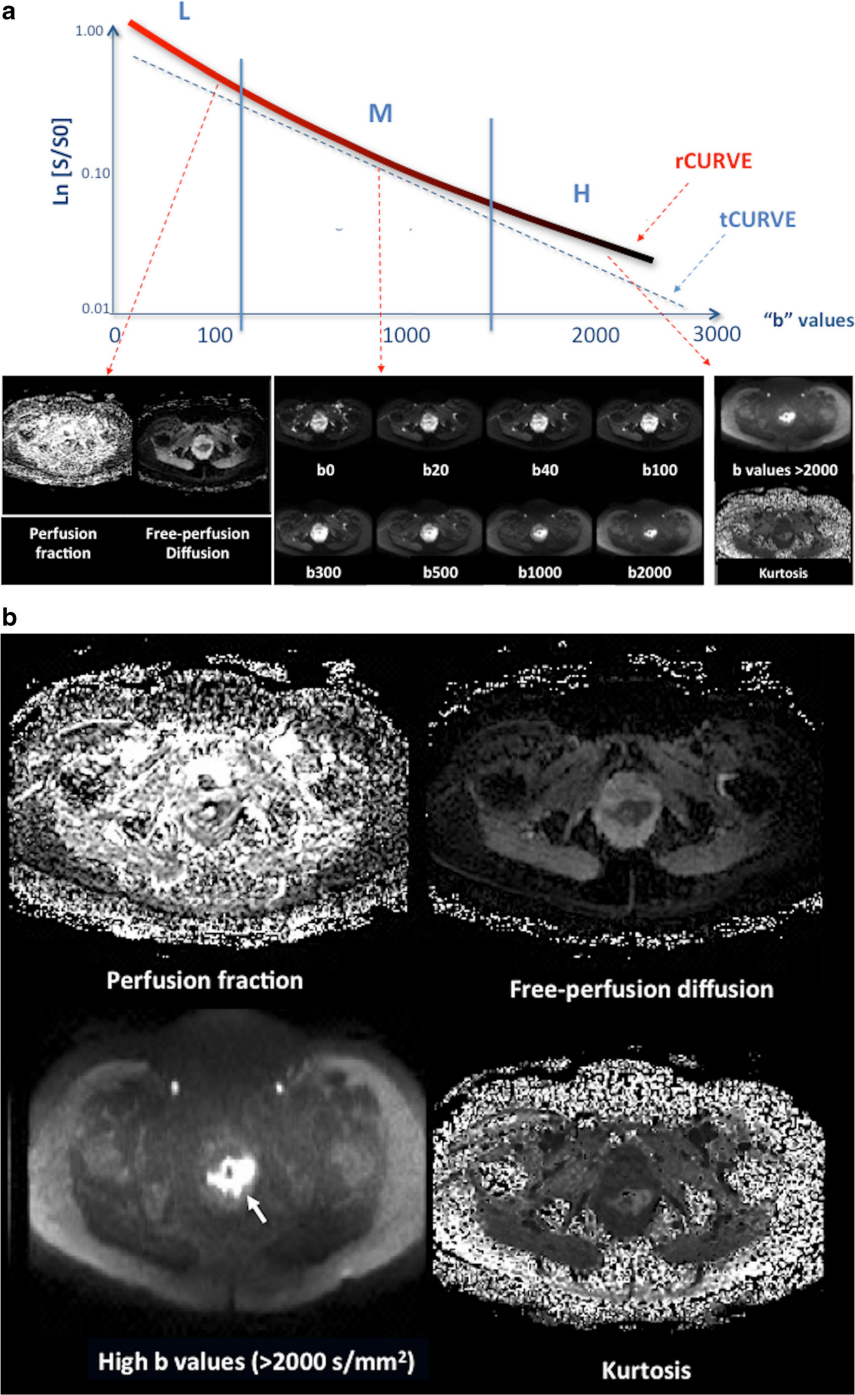
Diffusion analysis in a rectal tumour. Analysis of the relationship between signal attenuation in tissues with different b-values. Theorical (tCURVE) and real (rCURVE) curves of signal decay are different. (**a**) At low b-values (L), the signal is suppressed by small diffusion weightings (e.g., b value ≤ 100 s/mm^2^), which can be attributed to microcapillary perfusion, and intravoxel incoherent motion (IVIM) analysis may quantify the diffusion and perfusion effects separately. At medium b values (M) (100-1000 s/mm^2^), signal decay usually shows a Gaussian diffusion behavior, which would result in linear decay of the natural logarithm of the DWI signal intensity (SI) as the b-value increases, and subsequent quantification can be performed using a mono exponential analysis. On its part, at high b-values (e.g., >2000 s/mm^2^) (H), diffusion restriction is mainly secondary to cellularity, and quantification of non-gaussianity for water diffusion is possible based on diffusional kurtosis imaging (DKI), which may evaluate tissue structure that creates diffusion barriers and compartments. Tumours usually show an increased kurtosis. Pictured is an example of bi-exponential analysis of diffusion in a rectal tumour (arrow) (**b**), which allows the calculation of perfusion-related parameters, including the perfusion fraction (f) and perfusion-free diffusion (D) (top row), and an image of tumour kurtosis (bottom right)

DWI can be also used for assessing tumour vascularization. The perfusion component dominates the signal decay at low b-values and can be assessed using intravoxel incoherent motion (IVIM) analysis (Fig. 5). This feature enables differentiation between the perfusion fraction (f) and perfusion-free diffusion (D). The IVIM-related parameters may be used in the non-invasive evaluation of tumour perfusion. Bäuerle et al. found that f correlated to the vascular area fraction (percentage of CD 31 positive-staining area) on histological evaluation in the normal rectum and in tumours [45]. However, this correlation or significant changes on f have not been evidenced after CRTP [45, 46], which restricts the clinical value of IVIM in CRC.

#### Perfusion CT

**Fig. 6:**
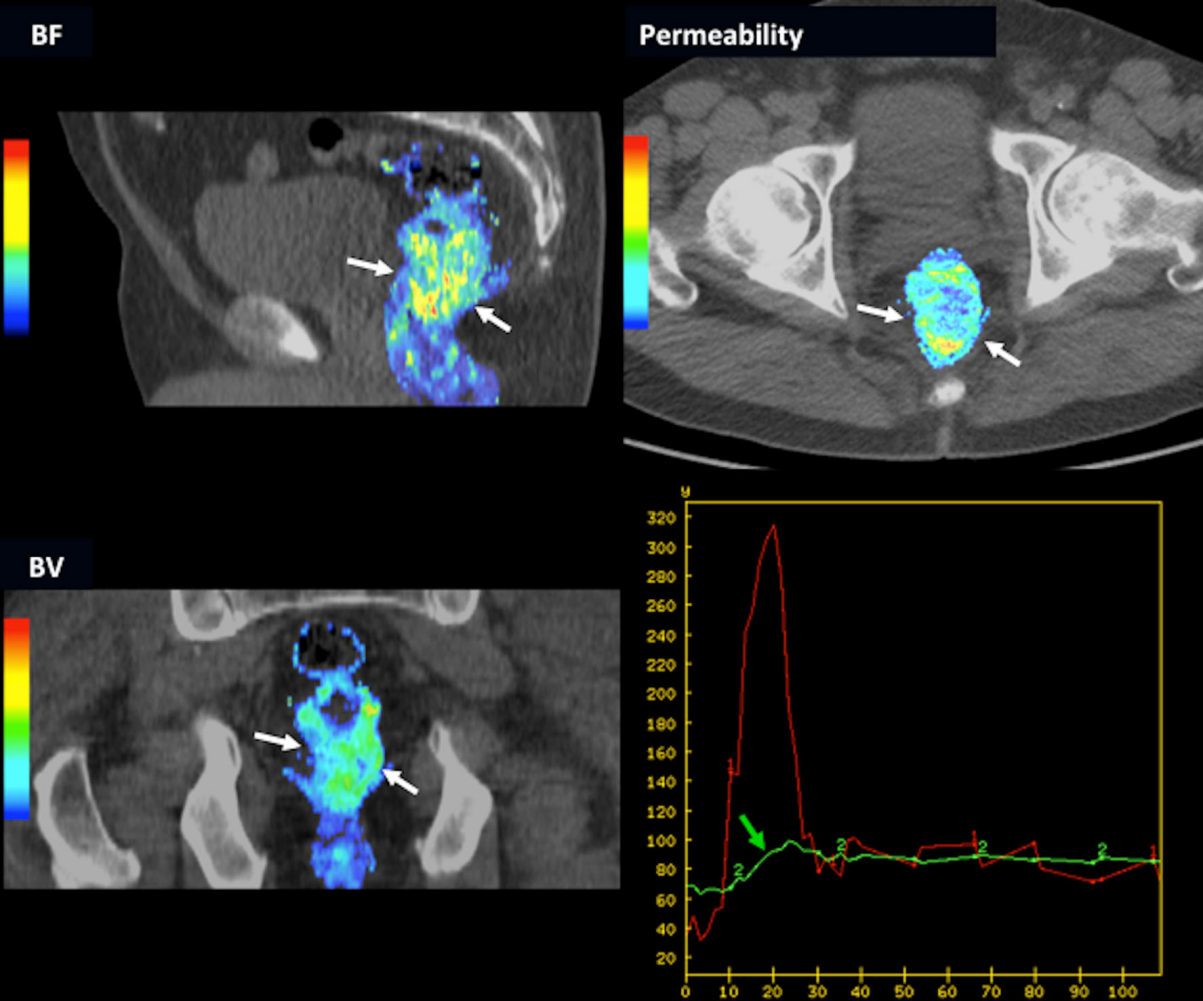
Volumetric perfusion CT multiplanar and multiparametric evaluation in a 62-year-old man with rectal cancer (white arrows). Parametric maps fused (50 % transparency) with CT images in different planes demonstrated increased values of perfusion-related parameters within the tumour: sagittal reformatted blood flow (BF) map, axial reformatted permeability map, and coronal reformatted blood volume (BV) map. Time-density curve of the tumour (bottom-right) demonstrated an enhancement curve type 3 (green arrow)

DCE-CT or perfusion CT (PCT) is an attractive technique for the evaluation of tumour vasculature based on the temporal change in tumour enhancement following intravenous iodinated contrast agent administration. Pharmacokinetic models allow obtaining qualitative data and quantitative parameters on tumour vascularization including blood flow (BF), blood volume (BV), mean transit time (MTT), permeability-surface area, or K^trans^ [34, 35] (Fig. 6). Tumour perfusion-related parameters (BF) may also distinguish the normal colonic wall from CRC (10–40 ml/min^−1^ 100 g^−1^ tissue vs. 50–200 ml/min^−1^ 100 g^−1^ tissue, respectively) [34]. Variations in tumour phenotyping and intratumoural heterogeneity may also be assessed based on the combination of perfusion-related parameters (Table 2) [34, 47], although the use of global mean values for perfusion parameters may underestimate the extent of spatial heterogeneity. In clinical practice, CT perfusion-related parameters may separate well, moderately, and poorly differentiated RC. Sun et al. reported that the mean BF was significantly different among well, moderately, and poorly differentiated groups (61.17 ± 17.97, 34.80 ± 13.06, and 22.24 ± 9.31 mL/minute/100 g, respectively) [48]; while Kim et al. evidenced that BF was higher in moderately differentiated CRC than well differentiated and poorly differentiated CRC [49] Although, certain limitations of these studies must be considered. First, the study sample size was small and second, there was no precise correlation between tumour ROI in perfusion CT and pathologic specimen after surgery. PCT parameters may also have a role as a potential prognostic biomarker in CRC. Hayano et al. evidenced that patients with more poorly perfused RCs (<40 ml/min^−1^ 100 g^−1^ tissue) had a poorer outcome [50]. Finally, PCT can also depict therapy-induced modifications in the vascularization of CRC [34, 35, 47, 51]. Changes in tumour vascularization depend on both the therapeutic mechanism of action and the timing of response evaluation. In this setting, neoadjuvant CRTP produces a decrease in perfusion parameters (reduction in BF around 40 %), although an early temporal increase can be depicted secondary to radiation-induced inflammatory changes [52]. In the case of CRC liver metastases treated with therapeutic regimens including antiangiogenic agents, several studies also have shown a reduction in BF and permeability after the therapy [53, 54].

**Table 2:** Biological correlation of perfusion-related parameters

BV and BF (often coupled)	Permeability	Biological interpretation
↑↑↑	↓ or ↔ or ↑	New vessel formation (increased perfusion and blood volume) with varying degrees of maturation
↓ or ↔ or ↑	↑↑↑	Poor perfusion with increased permeability (immature vessels), which usually represent a reaction to hypoxia
↑	↔ or ↓	Mature vasculature
↑	↔ or ↑	Inflammation +/− early fibrosis
↓	↓	Mature fibrotic areas (usually showing a progressive enhancement)
↓↓	↓↓	Poor vascularized areas
BF↓ relating to BV	----	Blood flow-blood volume mismatch, which usually represents hypoxia
+/− 0	+/− 0	Necrotic areas

Note—Data from literature reviews and personal experience and adapted from references 30,31, and 45

BV = blood volume, BF = blood flow

PCT is actually a robust technique on the basis of recent technological advances, including whole-tumour coverage, motion correction, noise reduction algorithms, etc. However, quality control is essential to enable CT quantification and efforts are needed toward a standardization of acquisition and data processing.

Finally, many features have to be considered that can affect the calculation of vascular parameters in both CT and MRI, including the conditions of signal production, the characteristics of the contrast agent, and the data analysis model. There are several important differences between DCE-MRI and PCT. The attenuation (expressed in Hounsfield units) is directly proportional to contrast agent concentration on CT. For its part, on MRI, the image intensities depend on many features (underlying native signal of the tissue or various parameters of the imaging sequences) adding complexity to analysis. Beside this, physiological explanation of some MRI-based quantitative parameters is complex, while PCT usually provides a series of more comprehensive parameters. CT offers a wider availability, but also shows potential limitations, including radiation dose, contraindications to iodinated contrast media, or the fixed axial plane of CT scanning. In the case of DCE-MRI, this technique is recommended over PCT for relatively young patients and offers a better signal-to-noise ratio, a stronger contrast uptake, and the absence of ionizing radiation [6–8, 31].

### Imaging tumour proliferation and cellularity in CRC

A basic characteristic of cancer is uncontrolled cell proliferation, which generally causes a greater cell density in tumour lesions.

#### Functional imaging of cellularity: Diffusion-weighted imaging in CRC

**Fig. 7:**
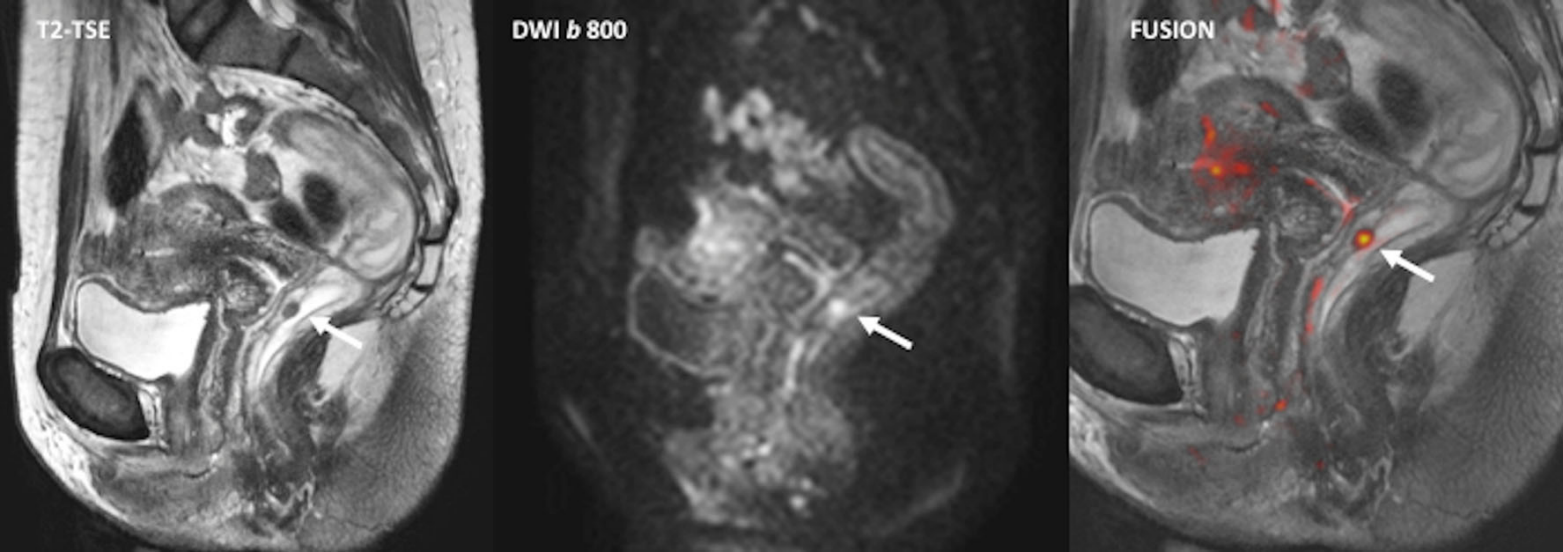
Diffusion for detecting colorectal tumours. A carcinoid tumour (white arrows) in a 44-year-old patient. Sagittal fast spin-echo T2-weighted image (left) and sagittal diffusion-weighted image with high b value (b = 800 s/mm^2^) (center) showed a rectal tumour nodule. Fused image (right) superimposing sagittal T2-weighted MR image and color-coded map derived from high-b-value diffusion-weighted image clearly delineated the tumour

To date, DWI is a basic technique in oncologic imaging [55]. Diffusion measures the random Brownian motion of water molecules within a voxel of tissue. The relationship between histology and diffusion is complex. DWI can mainly provide an indirect evaluation of cellularity and the integrity of cell membranes, but gland formation, perfusion, or cell death may also influence water diffusion. Besides, diffusion can be quantitatively assessed using the apparent diffusion coefficient (ADC) value. In CRC, this technique has shown to be of value for tumour detection, staging, prognosis, evaluation of response, and assessment of recurrence. DWI is a useful tool for detecting colorectal tumours. Ichikawa et al. reported a sensitivity and specificity of high-b-value DWI for detection of CRC of 91 % and 100 %, respectively [56] (Fig. 7). However, this study had some limitations, including a small study population and the fact that it did not include other benign conditions (i.e., inflammatory bowel disease), which could reduce its specificity. DWI may also improve tumour staging. This technique increases the sensitivity for detecting LNs. In a study by Heijnen et al., DWI detected 6 % more nodes than T2-weighted imaging [57]. Concerning the characterization of LNs, specificity and accuracy also increased after adding DWI to T2-weighted images, although the diagnostic accuracy of ADC for discriminating metastatic from non-metastatic LNs is only around 70 %, because subjective visual assessment cannot discriminate between benign and malignant nodes, as both display high DWI signals with increasing b-values, and the ADC values of malignant nodes have been shown to be only slightly lower than that of benign nodes—not enough to allow their discrimination.

**Fig. 8:**
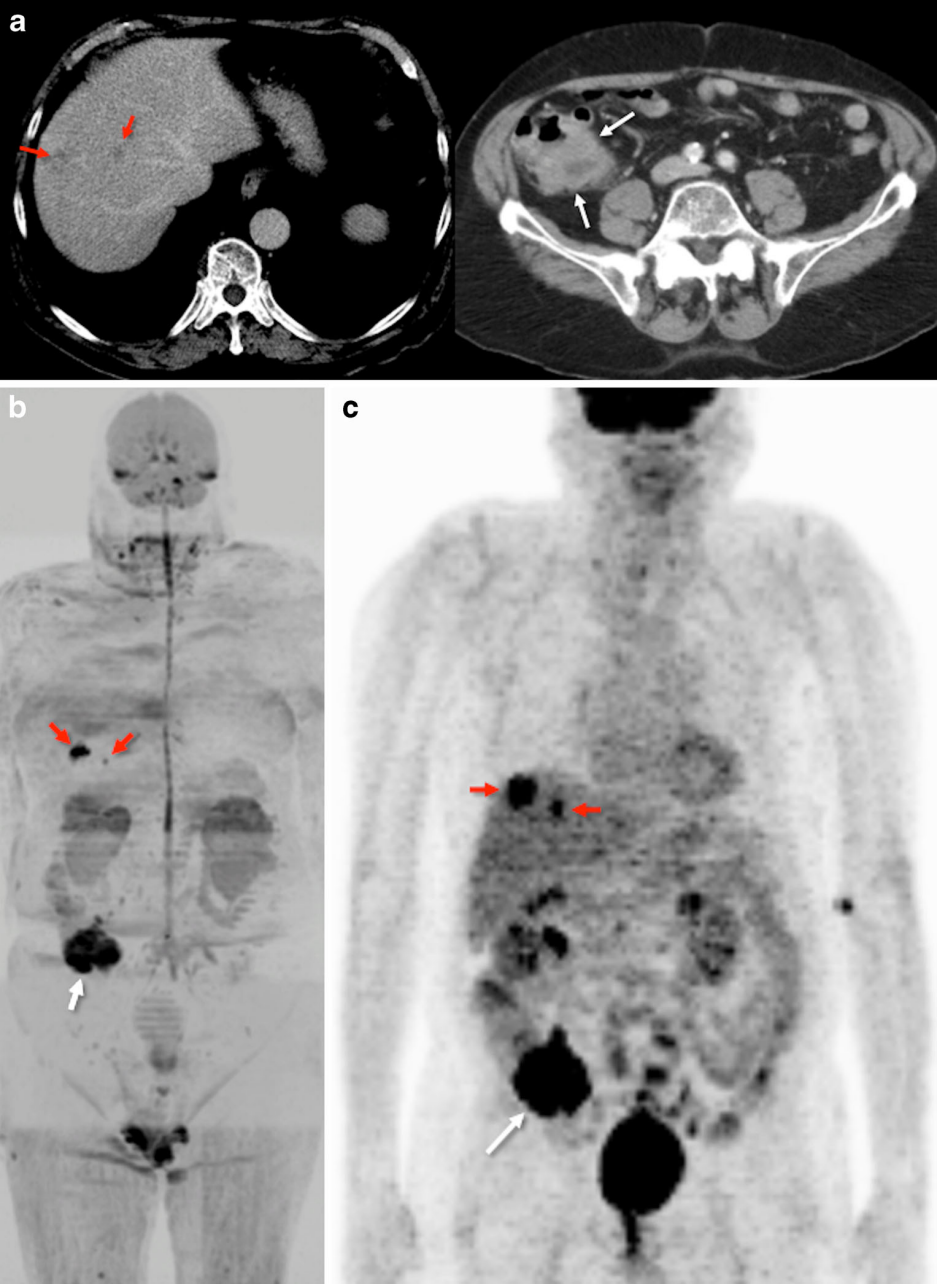
Whole-body diffusion imaging for tumour staging. Conventional portal-phase contrast-enhanced images (**a**) demonstrated two liver metastases (left – red arrows) and a tumour in the cecum (right - white arrow). Whole-body-diffusion-weighted image with inverted gray scale (**b**) depicted both focal liver lesions (red arrows) and the tumour in the cecum (white arrow) that showed restriction of diffusion. ^18^F-FDG-PET image (**c**) evidenced similar findings

Besides this, complete radio-pathological correlation was not possible in published studies, and a good correlation between anatomical and DWI sequences was difficult to achieve due to partial volume effects (based on the different voxel size used in both sequences) [58, 59]. The use of whole-body (WB)-DWI may be an attractive alternative for staging CRC (Fig. 8). A small study evidenced that the overall sensitivity of WB-DWI as a single modality for the detection of malignant lesions was 81 %. All primary CRC were detected, included 77 % of the liver metastases, 72 % of the distant nodal metastases, and 75 % of the lung metastases [60]. Further, in a study including 28 gastrointestinal cancers (23 CRC), Gong et al. found no statistically significant difference in the overall diagnostic performances of PET-CT (accuracy 98.9 %; sensitivity 95.2 %; specificity 99.8 %; PPV 98.9 %; NPV 98.9 %) and WB-DWI (accuracy 95.9 %; sensitivity 81.7 %; specificity 99.1 %; PPV 95.0 %; NPV 96.1 %) for the initial diagnosis or post-operative follow-up in detecting distant metastases or recurrence [61].

**Fig. 9:**
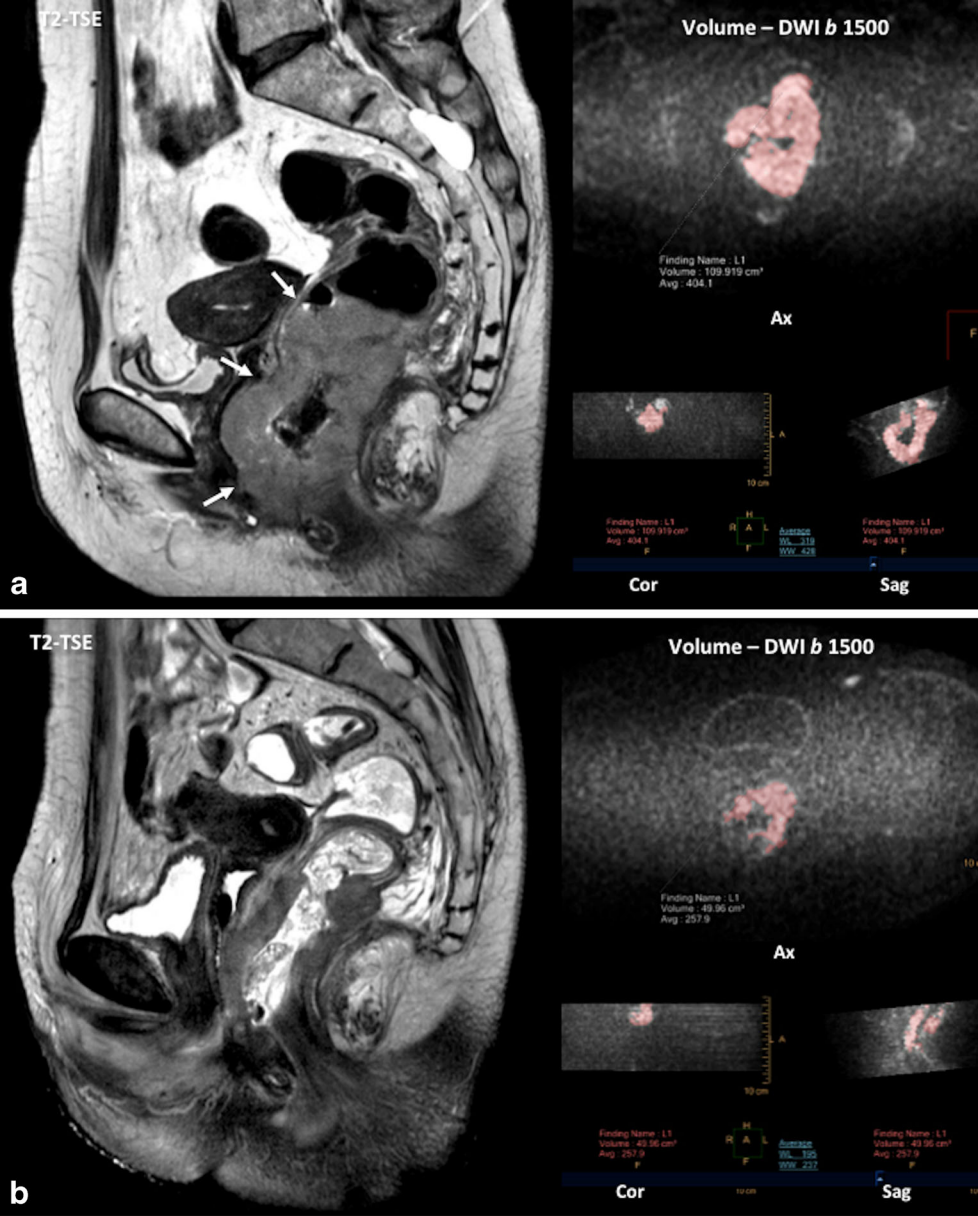

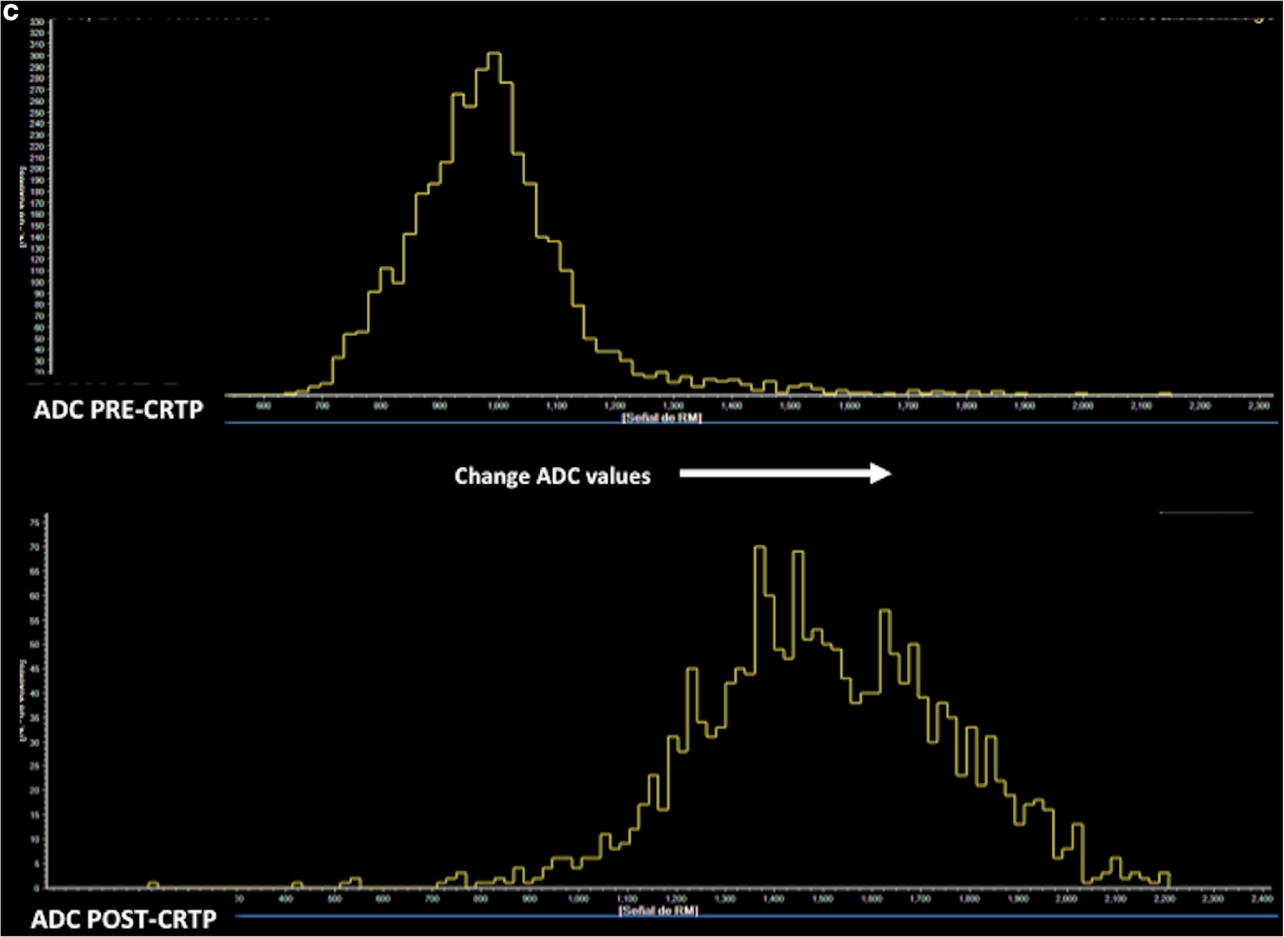
Diffusion for the evaluation of tumour response. Pretherapy (**a**) sagittal T2-TSE image and volumetry (volume = 109.91 cc) based on diffusion (b = 1500 s/mm^2^) image showed a big rectal cancer with invasion of the anal canal (white arrows). Post-therapy (**b**) volume was reduced (volume = 42.96 cc). Histogram analysis© pre and post-therapy of the apparent diffusion coefficient (ADC) also demonstrated increased ADC values

The role of diffusion as a prognostic or predictive tool has been also evaluated. In this setting, RC with lower ADC values was associated with more aggressive tumour behavior [62, 63]. Beside this, diffusion may predict RC response to neoadjuvant CRTP [64, 65]. A significant correlation between tumour volume reduction and pre-CRTP ADC values has been reported [64]. Pre-CRTP ADC of the histopathological responders was significantly lower than that of the histopathological non-responders and the change of ADC of the responders was significantly higher. Concerning tumour response evaluation, patients with a pCR after CRTP always have a better prognosis than those with other TRG grades. Increases in ADC values occur within 3–7 days in responding patients treated with CRTP [66]. Preliminary results indicate that DWI improves the diagnostic performance of MRI to detect early tumour response and to predict mesorectal fascia tumour clearance [67]. DWI, DW-MR-volumetry, and ADC histogram analysis are significantly more accurate than T2-weighted images in assessing tumour response [68–70] (Fig. 9). However, MRI showed heterogeneous results of diagnostic performances for restaging RC after CRTP, although better results were demonstrated when DWI was included [71]. A meta-analysis including 16 studies and 826 patients determined that the changes between the pre- and post-ADC are good predictors of a pCR, but some misjudgments remain, because DWI cannot reliably microscopically discriminate residual viable tumour cells from fibrosis, which can cause a considerable overlap of the ADC values between a pCR and near-pCR. Furthermore, DWI sensitivity is low, mainly due to the erroneous interpretation of high signals in ‘normal’ post-treatment rectal walls as residual tumour [72, 73]. These data are in agreement with a systematic review of the role of imaging (including DWI and fluorodeoxyglucose (^18^F-FDG) positron emission tomography (PET-CT) in the re-staging of RC after CRTP, which suggests that the major strength of imaging lies in the identification of non-responders. Both DWI and ^18^F-FDG PET-CT are not actually accurate enough to safely select patients for possible organ-sparing strategies [70]. In the case of tumour recurrence, a study by Lambregts et al. using DWI for diagnosing local tumour regrowth during follow-up of organ preservation treatment after CRTP for RC evidenced that although there was no overall improvement in diagnostic performance in terms of AUC, DWI improved the sensitivity of MRI for diagnosing local tumour regrowth and lowered the rate of equivocal MR exams [73].

Finally, we must consider that the standard mono-exponential analysis of DWI assumes a Gaussian behavior of water diffusion. However, in many biological tissues, the presence of barriers (e.g., cell membranes and intracellular organelles) and compartments (intracellular, intravascular, and EES) alter the water diffusion process so that it is no longer Gaussian. The kurtosis is a dimensionless statistical metric for quantifying the non-gaussianity of diffusion. A large diffusional kurtosis suggests a high degree of diffusional heterogeneity and microstructural complexity, which is usually the case of tumour lesions (Fig. 5) [74]. Preliminary results in body imaging open the consideration of a future role for this imaging technique in RC [74].

#### Molecular imaging of tumour proliferation with PET

^18^F-3-deoxy-3-fluorothymidine (FLT) is a radiotracer, which allows the evaluation of cellular proliferation. Nonetheless, FLT-PET evidenced a limited value in CRC, demonstrating less sensitivity than ^18^F-FDG-PET for the detection of pathologic LNs or liver metastases in CRC [75, 76].

### Tumour metabolism in CRC

Tumour proliferation needs nutrients, energy, and biosynthetic activity. This feature is responsible for the metabolic reprogramming associated with cancer. Imaging techniques allow assessment of the status of the altered metabolic pathways in CRC.

#### Imaging energetic metabolism with ^18^F-FDG-PET

**Fig. 10:**
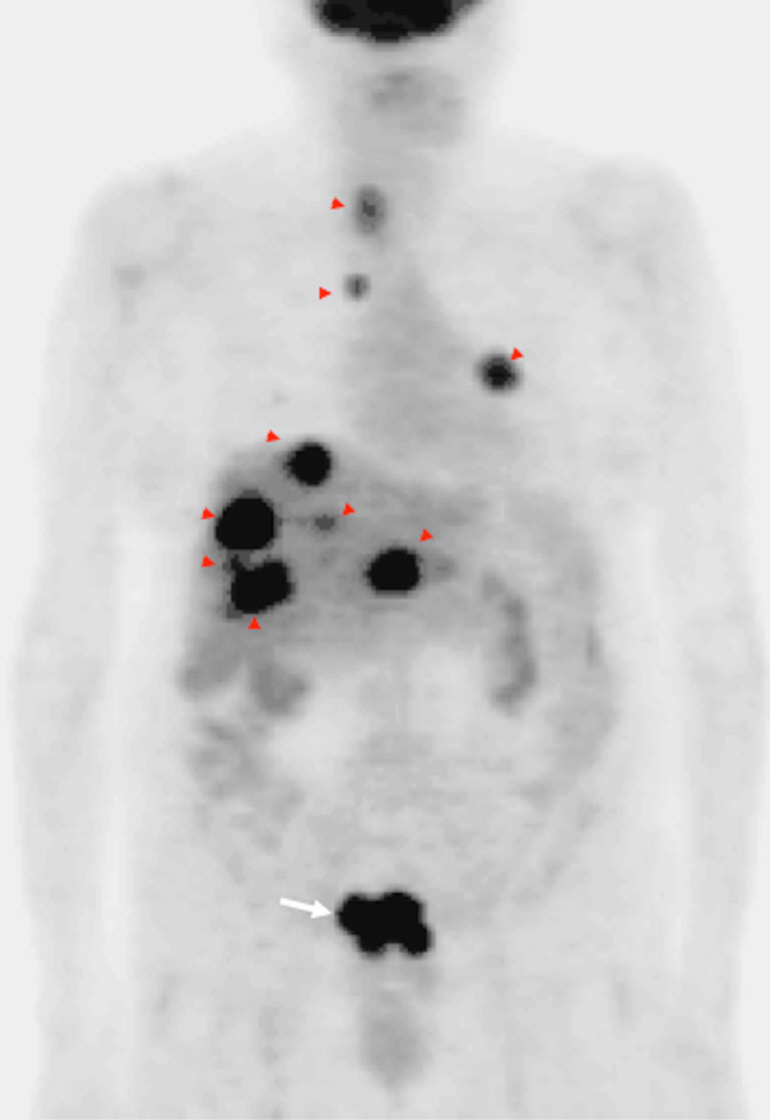
^18^F-FDG-PET in staging rectal cancer. Coronal PET image evidenced a focal area of uptake in the pelvis corresponding to the primary tumour (white arrow). PET also demonstrated multiple liver, lung, and lymph-node metastases (red arrowheads)

**Fig. 11:**
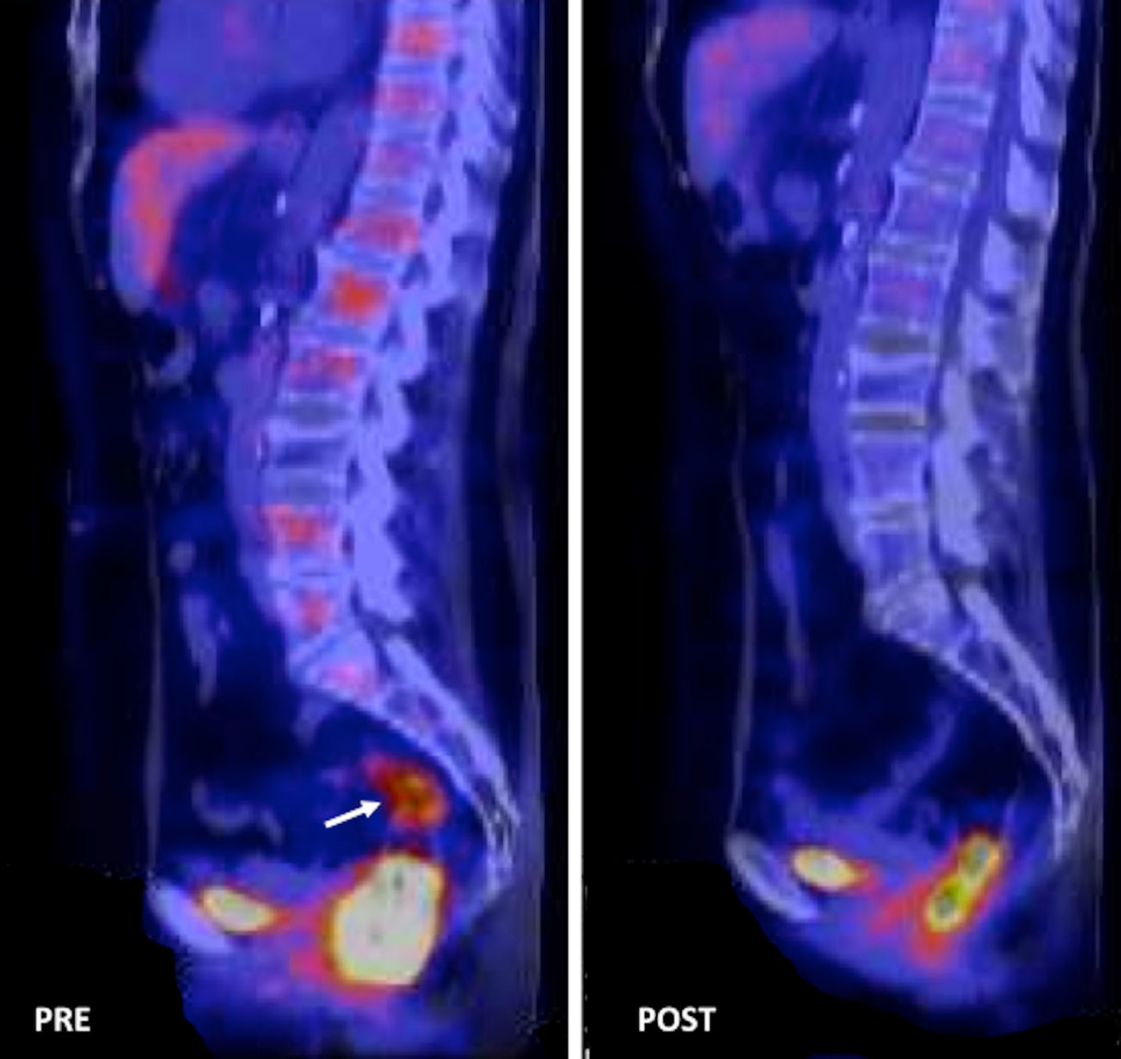
^18^F-FDG-PET for evaluating response to therapy. Changes secondary to therapy in a patient with rectal cancer treated with chemoradiation. Sagittal ^18^F-FDG-PET-CT image pretherapy (left) demonstrated an FDG-avid rectal mass (white arrow). Post-neoadjuvant chemoradiotherapy ^18^F-FDG-PET-CT image evidenced a complete tumour response (Courtesy JM Llamas- Elvira, MD. Department of Nuclear Medicine, Hospital Universitario Virgen de las Nieves, Granada, Spain)

A well-known energy metabolism alteration in tumour cells is an increased glycolytic capacity, even in the presence of a high O_2_ concentration, a process named aerobic glycolysis. Proliferating tumour cells generally consume glucose at a high rate. This increased glucose uptake is the basis for clinical PET imaging in tumours. PET-CT is an established clinical technique for the management of CRC patients, which may have an impact on changing patient management strategies with its evolving role in diagnosis, radiation therapy planning, prediction of response, and therapy assessment [77–80]. PET-CT may upstage a significant proportion the patients in RC by identifying unsuspected systemic or LN metastases [77, 78]. Nonetheless, routine PET for the initial staging of CRC is not an established indication of the technique. PET may also help to guide decisions concerning metastasectomy for patients with CRC by excluding unresectable metastatic disease [77]. Some papers support the accuracy of PET in predicting pCR, while others dispute its utility [70, 81–83] (Figs. 10 and 11). However, PET will likely not have the ability to detect patients with pathologic evidence of very few tumour cells in fibrotic tissue in the surgical specimen after CRTP [70]. A meta-analysis established that complete metabolic response on ^18^F-FDG-PET data after preoperative CRT is predictive of OS in RC [84]. Finally, PET is also more sensitive than conventional imaging (CT) in detecting tumour relapse. CT scans were positive in 82 % of patients and ^18^F-FDG-PET-CT in 98 % of patients [85]. However, several limitations of PET for the evaluation of CRC must be considered. ^18^F-FDG uptake is dependent on tumour grade and histological type, and PET shows a limited and a poor spatial resolution (missing small lesions).

PET-MRI has recently become available for clinical use. Combined anatomic and functional capabilities of MR imaging and the metabolic information of PET provide new insight into tumour phenotypes at a lower radiation dose than a PET-CT. Current literature is sparse concerning the role of PET-MRI in CRC [86]. Perhaps this hybrid technique might prove valuable in offering an increased confidence in the evaluation of liver lesions and residual masses after treatment, and improving identification of LNs.

#### MR spectroscopy for evaluating metabolism

**Fig. 12:**
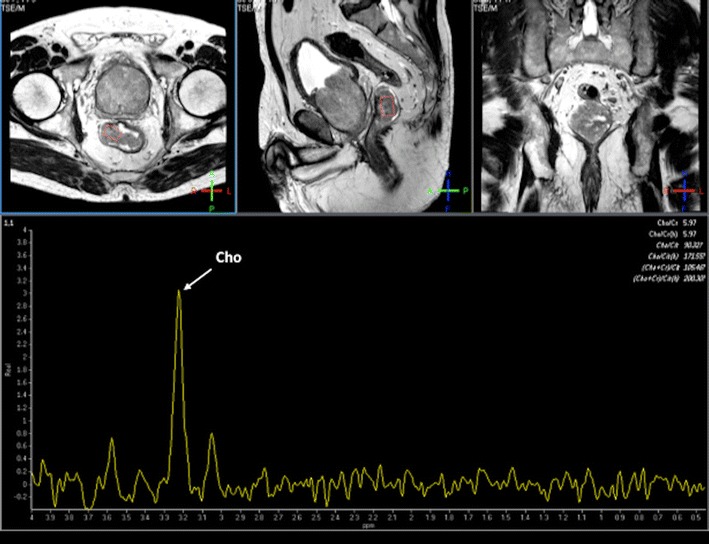
MR spectroscopy of rectal polyp. Proton MRSI of a rectal polyp. Axial (upper row), sagittal (middle), and coronal (right) images obtained by T2-weighted turbo spin-echo imaging. Spectra obtained by univoxel spectroscopy at long echo time (TE = 135 ms) showed an increased level of choline (Cho) peak at 3.2 ppm

There has been scarce literature published on the use of proton (^1^H) MR spectroscopy (MRS) in CRC. Kim et al. demonstrated that RC mainly showed elevated choline (Cho) at 3.2 ppm and lipids (Lip) peaks at 1.3 ppm on MRS (Fig. 12). After CRTP, the Cho peak disappeared, resulting in only the Lip peak [87]. MRS has also been found of interest in the diagnosis of postsurgical recurrence of RC. An increase of residual Lip peaks in the postsurgical bed suggests tumour recurrence, while lower Lip peaks are present in scarring postoperative fibrosis [88]. However, MRS shows no clear clinical application in CRC because MRS evaluation of these tumours is technically demanding, not only for bowel movement but also for the presence of multiple interfaces between air, rectal wall, and perirectal fat that may result in an inhomogeneous magnetic field and poor spectra quality.

### Imaging oxygenation and hypoxia in CRC

**Fig. 13:**
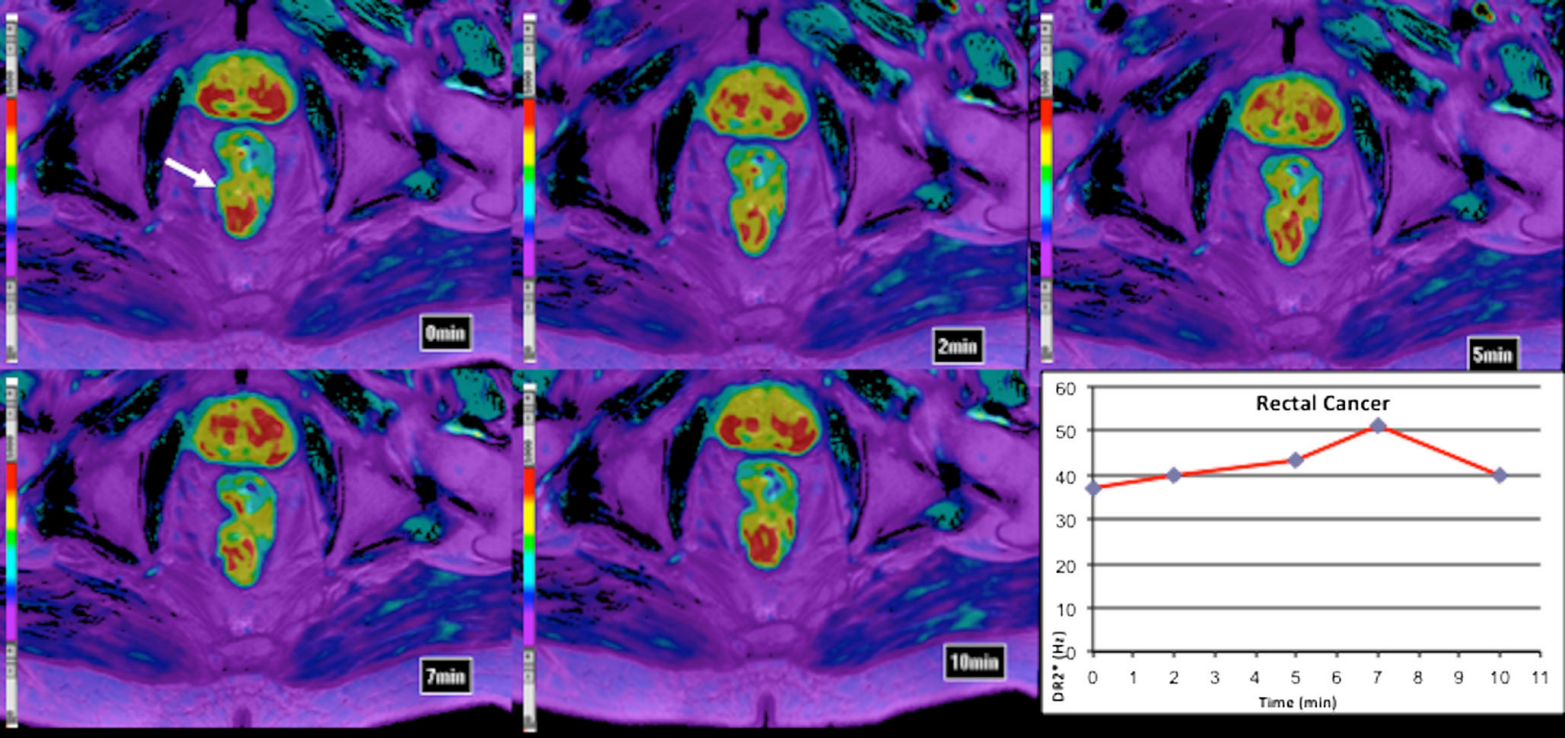
Tumour hypoxia. BOLD images (fusion of T2 and T2*) pre- and following the administration of oxygen may demonstrate a mucinous adenocarcinoma of the rectum. In basal conditions, the tumour showed high signal on T2* map. After O_2_ administration, the signal decreased with an ascending curve of ΔR2* (red line), which is related to tumour radioresistance

Hypoxia is considered to be an important mediator of malignant disease progression that has an important role in predicting the response to radiotherapy and an impact on patient prognosis and survival in CRC. To our knowledge, imaging evaluation of hypoxia in CRC has been scarce in published literature. Blood oxygenation level-dependent (BOLD)-MRI may provide a non-invasive means of assessing in-vivo tumour oxygenation based on endogenous deoxyhemoglobin as a contrast agent (Fig. 13). However, BOLD-MRI shows several limitations that need to be considered. First, no correlation between BOLD-MRI measurements and hypoxic markers has been published in CRC. Second, this technique is more likely to reflect acute tissue hypoxia (perfusion-related and often transient) than chronic (caused by increased oxygen diffusion distances due to tumour expansion) hypoxia and requires the simultaneous assessment of the functionality of tumour vasculature. And third, motion and susceptibility artifacts limit the clinical application of BOLD-MRI in CRC [89]. There is also a limited experience with the PET evaluation of hypoxia in CRC using hypoxia-related radiotracers in CRC [75].

### Functional imaging of lymph nodes

**Fig. 14:**
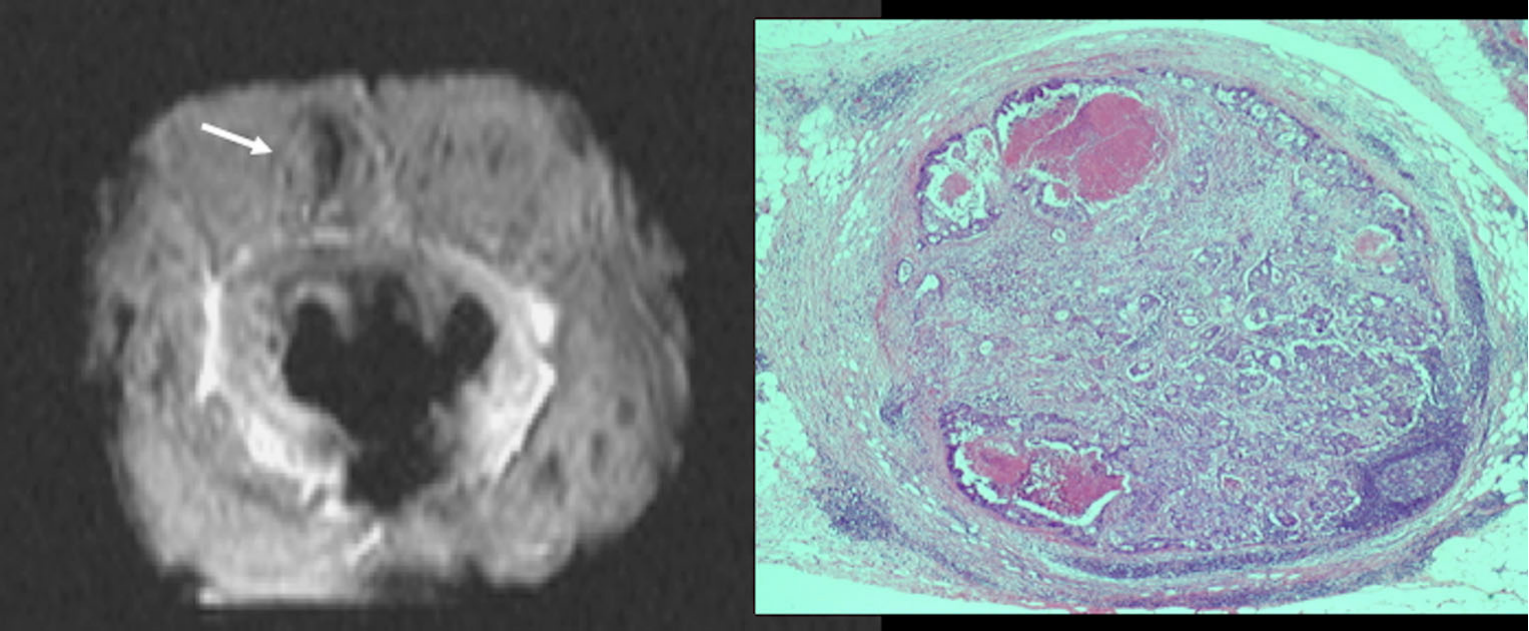
MR lymphography. Metastatic 6-mm node (white arrow) on T2*-weighted high-resolution specimen MRI with the histopathological correlation (right) in a patient with a pT3 pN1 moderately differentiated adenocarcinoma

Nodal metastases are one of the most significant indicators of local recurrence and cancer-specific mortality in RC patients, and influence the determination of surgical and adjuvant treatments. CT, MRI, and endorectal ultrasound lacked sufficient accuracy to identify metastatic LNs with sensitivities and specificities in the 55–78 % range. Apart from this, histological data show that up to 45 % of nodal metastases in RC are ≤4 mm, which increases the difficulty of the accurate characterization of LNs. The development of ultrasmall iron oxide particle (USPIO) contrast agents opened the possibility of performing MR lymphography (MRL) [90]. The contrast agent shows a specific cellular uptake by the macrophages in normally functioning nodes. Macrophage sequestration within normal LNs causes decreases in nodal signal intensity (SI) on susceptibility-weighted (T2*) MRI, whereas infiltrated LNs do not have macrophages and cannot take up the contrast agent (Fig. 14). MRL has demonstrated a sensitivity of 93 % and a specificity of 96 % for nodal staging in RC [91]. However, several limitations must be considered when we evaluated these data. First, MRI has a limited spatial resolution, meaning that only nodes that could be directly co-located between pre-operative in-vivo images and histopathology analysis were evaluated in published papers, thus excluding many other LNs (seen at pathology but not by MRI, and vice versa). This fact necessarily introduces a bias toward the assessment of larger LNs, which are naturally more visible by imaging. Second, an overlap of SI between benign and malignant LNs has been reported due to different features, including partial volume and lipomatosis. Finally, the evaluation of numerous LNs is a complex and time-consuming activity. Perhaps the use of USPIO combined with DWI may simplify the process and allow for more accurate detection of nodal metastases [92]. However, to date, the use of USPIO agents in clinical practice is not possible.

The use of gadofosveset (a gadolinium-based MRI contrast agent, which acts as a blood pool agent) can also significantly improve the diagnostic performance to discriminate between benign and metastatic LNs in RC. The intravenous administration of this contrast media results in a selective uptake of contrast in benign LNs, which causes an increase in signal on T1-weighted images, and enhances the chemical shift artfact around the border of the nodes [93].

## Multiparametric evaluation of CRC

**Fig. 15:**
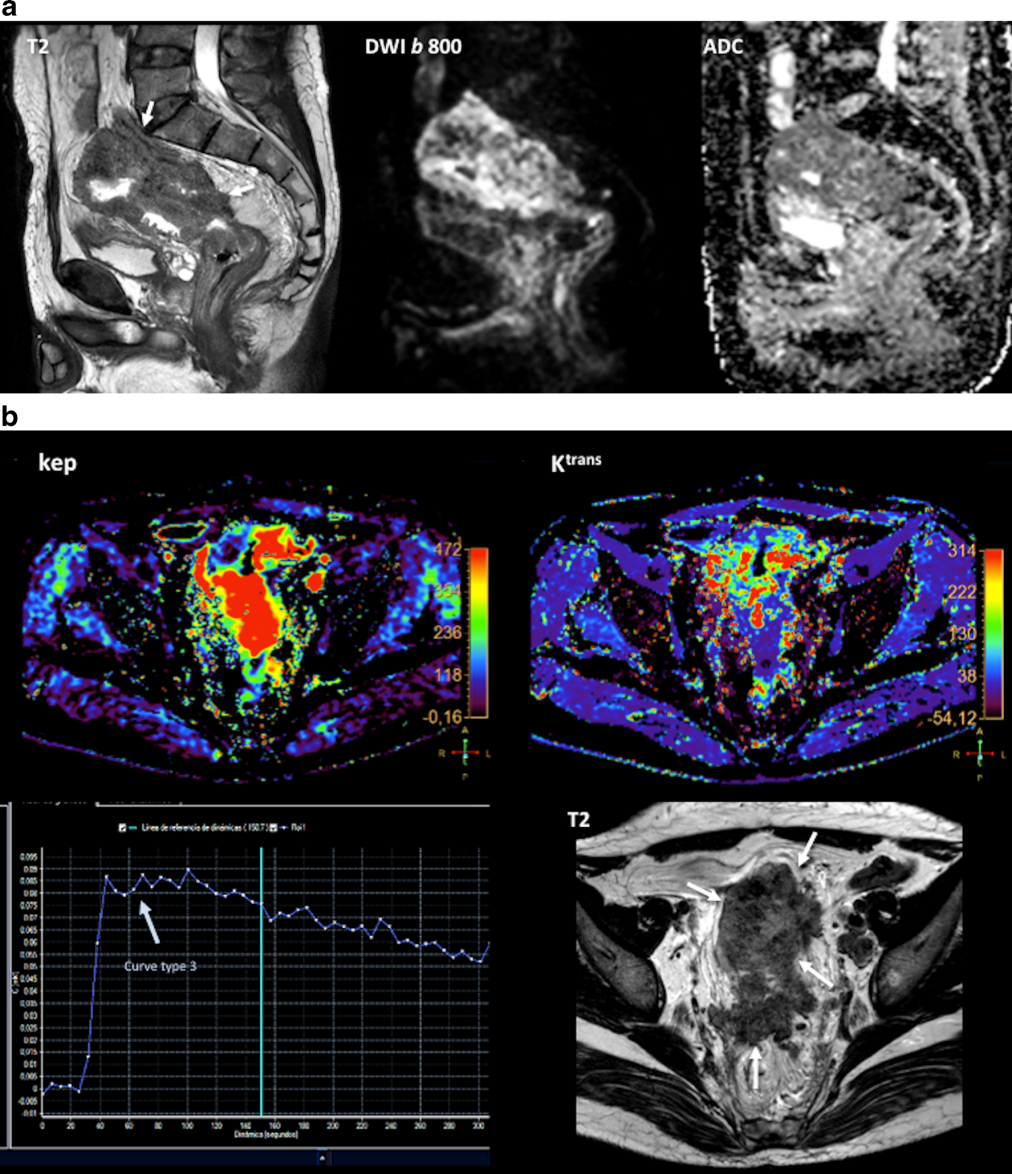

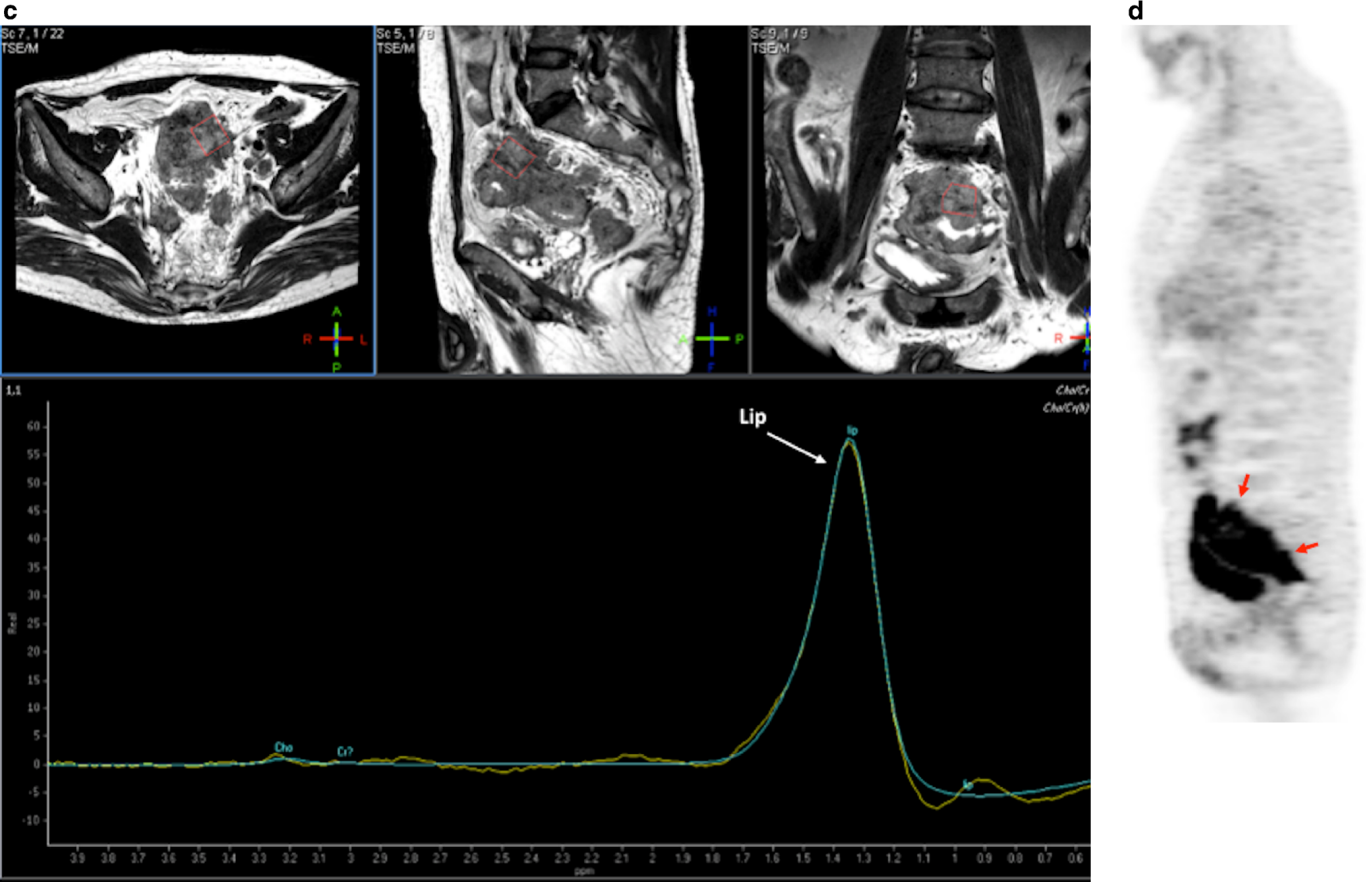
Multiparametric evaluation of a 64-year-old patient with rectal cancer, pre-therapy. (**a**) Sagittal fast spin-echo T2-weighted image (left) and sagittal diffusion-weighted image with high b-value (b = 800 s/mm^2^) (center) and ADC map showed a big rectal tumour with restricted diffusion. (**b**) Perfusion MRI-related parametric maps (transfer constant, K^trans^ and return constant, k_ep_), gadolinium concentration/time curve of the tumour evidenced increased perfusion within the tumour and a type 3 curve. (**c**) MR spectroscopy depicted an increased lipids peak. (**d**) Sagittal ^18^F-FDG-PET image evidenced an increased uptake of glucose in the tumour (red arrows)

**Fig. 16:**
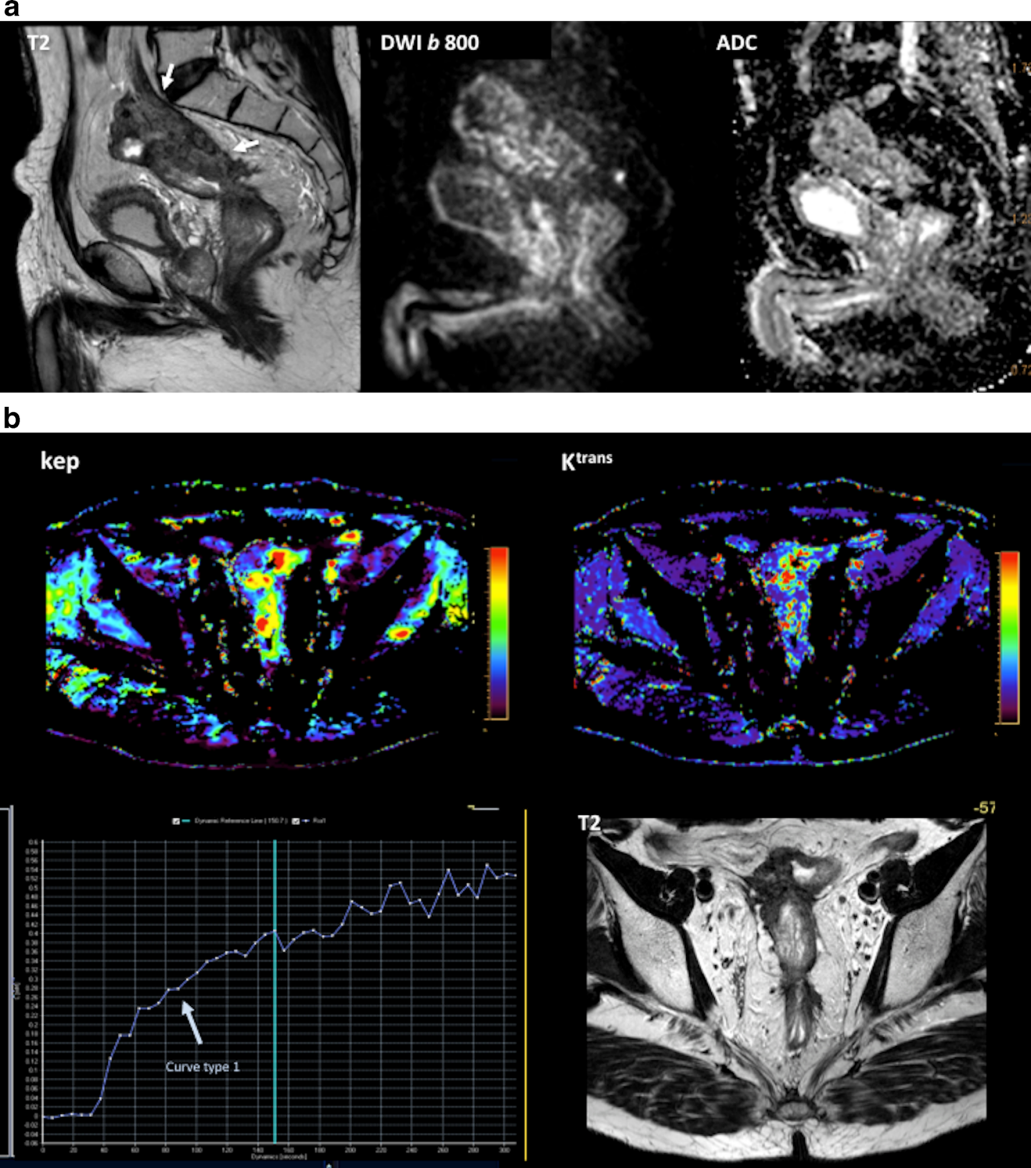

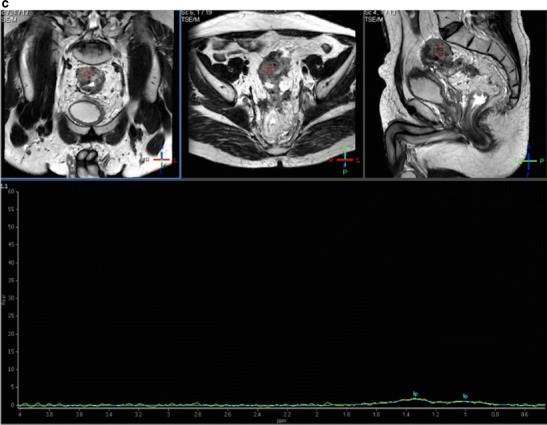

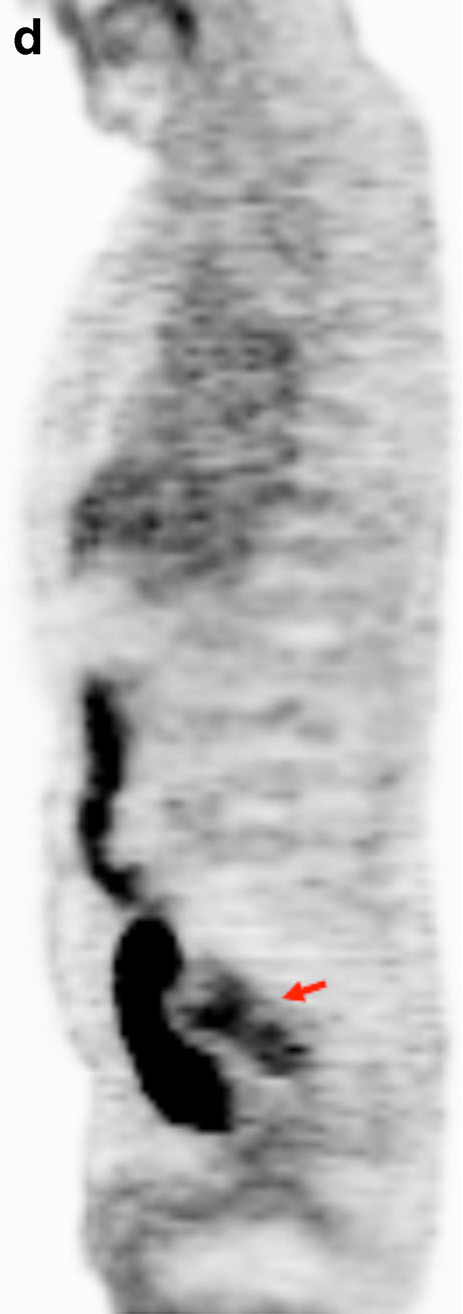
Multiparametric evaluation of a 64-year-old patient with rectal cancer, post-therapy. (**a**) Sagittal fast spin-echo T2-weighted image (left), sagittal diffusion-weighted image with high b-value (b = 800 s/mm^2^) (center), and ADC map showed a reduction of tumour volume and increased ADC values. (**b**) Perfusion MRI-related parametric maps (transfer constant, K^trans^ and return constant, kep) and gadolinium concentration/time curve of the tumour evidenced decreased perfusion within the tumour and a change in the type of curve (type 1 curve). (**c**) MR spectroscopy did not depict any metabolite peak. (**d**) Sagittal ^18^F-FDG-PET image evidenced a decreased uptake of glucose in the tumour (red arrow). All these features indicated partial tumour response

Combining the anatomical resolution of imaging with functional (such as DWI and DCE imaging) and molecular (PET) techniques may offer additional information about treatment of tumour microenvironments in different clinical scenarios, including staging, tumour characterization, prediction of response to treatment, and response evaluation [94–101]. The use of these techniques allow for the quantitative evaluation of tumour phenotype, including changes in tumour biology that occur after therapy (Figs. 15 and 16). There has been a very limited use of the multiparametric/multimodality approach in CRC. These studies are mainly confined to experimental settings, and most of them have been single-center studies with small numbers of patients. The relationship between different parameters may explain tumour biological features. In this setting, CRC with a low-flow/high-metabolism phenotype demonstrated higher vascular endothelial growth factor (VEGF) expression and may reflect a more angiogenic and aggressive phenotype [98]. Fischer et al. reported that changes in the flow-metabolic phenotype (blood flow × maximum standardized uptake values (SUV)) of RC after CRT showed high accuracy for the prediction of histopathological response to CRTP (AUC 0.955, 95 % confidence interval 0.833-1.000) using a cut-off value of −75 % [101]. However, at present, there is no standardized imaging protocol for multiparametric imaging evaluation in CRC patients, which complicates its clinical implementation.

## Conclusion

In conclusion, advanced imaging techniques offer great opportunities in the evaluation of patients with CRC. The introduction of functional and molecular imaging techniques in clinical practice allows for the assessment of tumour hallmarks and tumour heterogeneity, which may change the management and therefore the prognosis of patients.

## Abbreviations

(3D): 3-dimensional
(ADC): Apparent diffusion coefficient
(AUC): Area under the curve
(BF): Blood flow
(BV): Blood volume
(BOLD): Blood oxygenation level dependent
(CTP): Chemotherapy
(CRTP): Chemoradiotherapy
(Cho): Choline
(CRC): Colorectal cancer
(pCR): Complete pathologic response
(CT): Computed tomography
(CTC): Computed tomographic colonography
(DW): Diffusion-weighted
(DWI): Diffusion-weighted imaging
(DECT): Dual-energy computed tomography
(DCE): Dynamic contrast-enhanced
(EES): Extravascular extracellular space
(^18^F-FDG): Fluorodeoxyglucose
(FLT): ^18^F-3-deoxy-3-fluorothymidine
(FMI): Functional and molecular imaging
(IVIM): Intravoxel incoherent motion
(IC): Iodine concentration
(Lip): Lipids
(LNs): Lymph nodes
(MRI): Magnetic resonance imaging
(MRL): MR lymphography
(MRS): MR spectroscopy
(MTT): Mean transit time
(NPV): Negative predict value
(OS): Overall survival
(PCT): Perfusion CT
(f): Perfusion fraction
(D): Perfusion-free diffusion
(PP): Portal phase
(PPV): Positive predict value
(^1^H): Proton
(k_ep_): Rate constant
(PET): Positron emission tomography
(RC): Rectal cancer
(SUV): Standardized uptake values
(K^trans^): Transfer constant
(TVRR): Tumour volume reduction rate
(USPIO): Ultrasmall iron oxide particles
(VEGF): Vascular endothelial growth factor
(WB)-DWI: Whole-body diffusion-weighted imaging
